# Effects of transition on HIV and non-HIV services and health systems in Kenya: a mixed methods evaluation of donor transition

**DOI:** 10.1186/s12913-021-06451-y

**Published:** 2021-05-13

**Authors:** Daniela C. Rodríguez, Diwakar Mohan, Caroline Mackenzie, Jess Wilhelm, Ezinne Eze-Ajoku, Elizabeth Omondi, Mary Qiu, Sara Bennett

**Affiliations:** 1 Department of International Health, Johns Hopkins School of Public Health, 615 Wolfe Street, 8th Floor, Baltimore, MD 21205 USA; 2 Ipsos-Kenya, Nairobi, Kenya

**Keywords:** Donor transition, HIV, Health systems, Kenya, Mixed-methods evaluation, Sustainability

## Abstract

**Background:**

In 2015 the US President’s Emergency Plan for AIDS Relief (PEPFAR) initiated its Geographic Prioritization (GP) process whereby it prioritized high burden areas within countries, with the goal of more rapidly achieving the UNAIDS 90–90-90 targets. In Kenya, PEPFAR designated over 400 health facilities in Northeastern Kenya to be transitioned to government support (known as central support (CS)).

**Methods:**

We conducted a mixed methods evaluation exploring the effect of GP on health systems, and HIV and non-HIV service delivery in CS facilities. Quantitative data from a facility survey and health service delivery data were gathered and combined with data from two rounds of interviews and focus group discussions (FGDs) conducted at national and sub-national level to document the design and implementation of GP. The survey included 230 health facilities across 10 counties, and 59 interviews and 22 FGDs were conducted with government officials, health facility providers, patients, and civil society.

**Results:**

We found that PEPFAR moved quickly from announcing the GP to implementation. Despite extensive conversations between the US government and the Government of Kenya, there was little consultation with sub-national actors even though the country had recently undergone a major devolution process. Survey and qualitative data identified a number of effects from GP, including discontinuation of certain services, declines in quality and access to HIV care, loss of training and financial incentives for health workers, and disruption of laboratory testing. Despite these reports, service coverage had not been greatly affected; however, clinician strikes in the post-transition period were potential confounders.

**Conclusions:**

This study found similar effects to earlier research on transition and provides additional insights about internal country transitions, particularly in decentralized contexts. Aside from a need for longer planning periods and better communication and coordination, we raise concerns about transitions driven by epidemiological criteria without adaptation to the local context and their implication for priority-setting and HIV investments at the local level.

**Supplementary Information:**

The online version contains supplementary material available at 10.1186/s12913-021-06451-y.

## Background

The President’s Emergency Plan for AIDS Relief (PEPFAR), the US government’s (USG) flagship HIV support program, has been operating in Kenya since 2004. In 2014, PEPFAR invested USD 375 million to support treatment, care and prevention through technical assistance, capacity building, health systems strengthening, and direct support to scale-up services [1, 2]. Much of the support was channeled through implementing partners (IPs) that were contracted to serve a specific catchment area. Many PEPFAR IPs, particularly those funded via the US Agency for International Development (USAID), also provided support for maternal, neonatal, and child health (MNCH) services.

In 2015, PEPFAR initiated a Geographic Prioritization (GP) process aimed at reallocating investments within each PEPFAR country to the highest burden areas to accelerate achievement of UNAIDS’ 90–90-90 targets, namely increasing numbers of people living with HIV who know their serostatus, initiate treatment, and adhere to treatment to 90% each. PEPFAR-Kenya’s GP plan allocated counties into investment categories from most to least support (namely, Scale-up, Maintenance, Central Support). All facilities supported by PEPFAR IPs in Central Support (CS) counties would be transitioned to governmental support. Seven counties in Northeastern Kenya were assigned to CS (Garissa, Isiolo, Lamu, Mandera, Marsabit, Tana River, and Wajir) accounting for 1% of Kenya’s HIV burden (Maintenance accounted for 19%, Scale-up for 80%) [3].

HIV prevalence in Kenya has been hovering around 5.6% in the general population, with higher rates among women, young people, and key populations, such as sex workers or men who have sex with men, who have substantially higher prevalence rates (29 and 18%, respectively). HIV care is widely available across health facilities, and patients are starting on anti-retroviral therapy (ART) earlier [4]. However, there is considerable geographic variation in disease burden across Kenya with estimates ranging from over 25% prevalence in Homa Bay to 0.2% in Wajir [5]. Coverage of key HIV services in CS counties is considerably worse than elsewhere, with higher rates of mother-to-child transmission (up to 40% in Wajir), increasing infections among children, lower ART rates, and high ratings on the HIV Stigma Index [6]. PEPFAR IP support to CS counties was offered by two organizations, one whose portfolio covered Lamu county and another which covered the remaining six CS counties; both IPs also supported other non-CS counties. PEPFAR IPs support for HIV services included:

- Providing lay counselors for testing,
- Integration with MNCH and tuberculosis programs,
- Expert clients to promote treatment initiation and adherence,
- County-level planning and management, including for commodities, data reporting,
- Healthcare worker training and supervision, and
- Laboratory strengthening and networking.

IPs also provided support to MNCH, family planning, water and sanitation, and nutrition services. In total, 413 facilities were assigned to CS of which 404 (98%) received support from USAID, who commissioned this study to assess early effects of GP and make course corrections if necessary. Transition to CS was expected to take place by mid-2016 (see Table 5 in the Results for further details).

GP took place in a context of two major policy changes in Kenya. First, the health system went through widespread devolution of responsibilities in 2013 that coincided with a reorganization of the political structure of the country with 8 provinces divided into 47 counties. Responsibilities for health facility ownership and service provision shifted to county governments, while the national level retained stewardship responsibilities, such as policymaking and regulation [7]. In terms of the health system, all seven CS counties are considered “marginalized” counties eligible for equalization funds under devolution.^1^ Among them, only Lamu, Garissa and Isiolo allocated more than 20% of their budget to health in 2016/17, and across CS counties, more than 50% of health budgets are allocated to personnel costs [8, 9]. Doctors and nurses are not widely available in CS counties with Mandera, Tana River, and Wajir reporting less than 0.5 health workers per 10,000 population, Marsabit 4.4 per 10,000 population, and Isiolo and Lamu 7.3 and 10.4 per 10,000 population, respectively [10].

Second, specific to HIV, the National AIDS Control Council (NACC) and National AIDS/STD Control Programme (NASCOP) released the Kenya HIV Prevention Revolution Road Map in 2014, which outlined an approach focused on prioritization between counties based on HIV incidence (i.e., low, medium and high), among other strategies [11]. PEPFAR’s allocation of counties to different categories mostly aligned with that of the Government of Kenya (GOK), with the exception of Isiolo which was categorized as medium incidence in the GOK’s Road Map but was flagged for CS by PEPFAR.

The recent trend of donor transitions in global health [12] has been the focus of evaluations and research around the world. Transition evaluations for immunization and HIV programs have described the effects and lessons learned from transitions [13–15], including on program beneficiaries [16], and have identified lessons and guidance for future transitions, including assessing readiness and improved transition planning [14, 17–19]. Studies of USG transitions have been conducted in Latin America [20–22] and elsewhere for family planning (FP) [23], and in Bangladesh, Botswana, China, and Guyana for HIV [24].

This paper presents results from an evaluation of GP in facilities transitioning to CS in Kenya that documented the implementation of GP, identified changes in health systems, HIV and non-HIV services associated with GP, and factors that supported or hindered a successful transition. We included non-HIV services because most transitioning facilities were supported by USAID through mechanisms that might potentially also offer support to non-HIV services, primarily MNCH. To-date research is mixed on whether targeted HIV funding has positive effects on other services or crowds them out [25]. We believe this is the first evaluation of a transition to explicitly assess effects on both targeted and non-targeted services as well as health systems. This study was also conducted concurrently in Uganda with results published elsewhere [26, 27].

## Methods

**Fig. 1:**
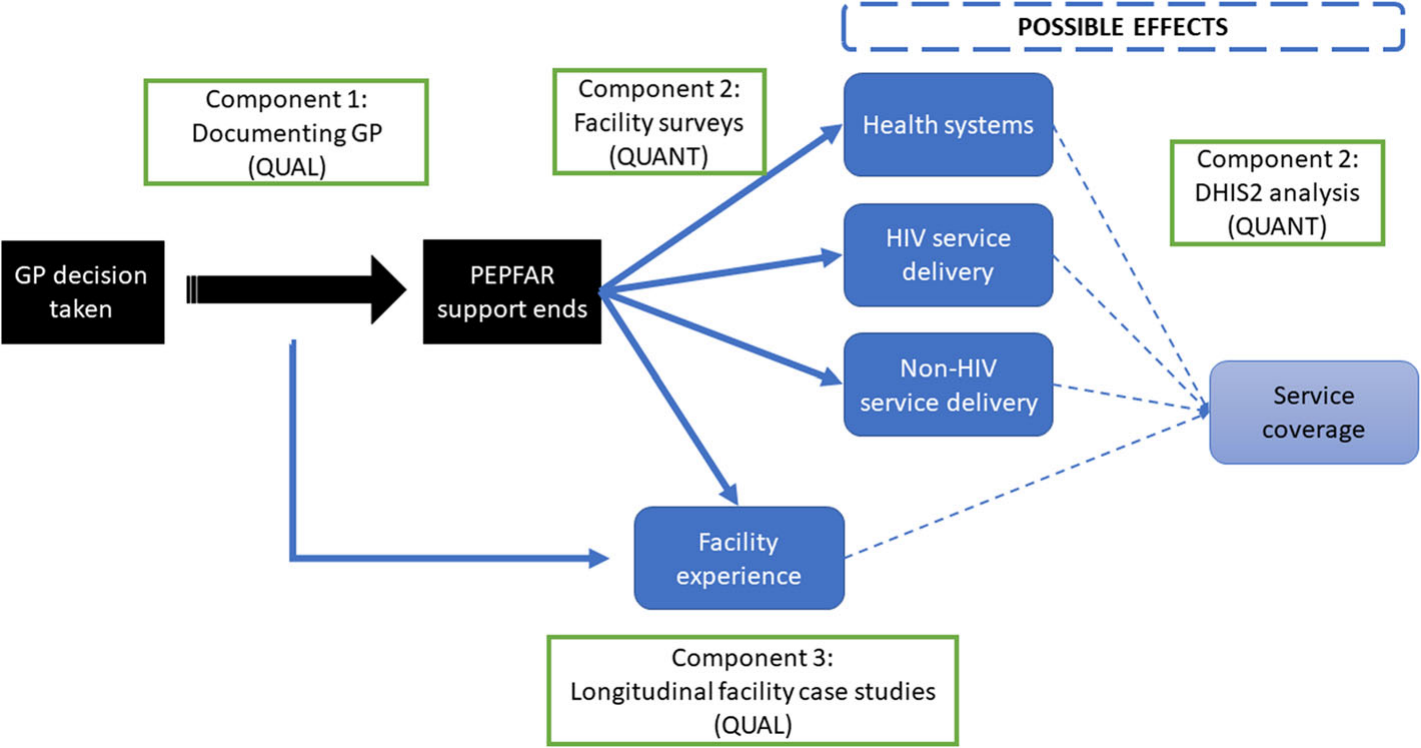
Study design

We employed a mixed methods approach for this study combining interviews, focus group discussions (FGDs), and a large-scale facility survey across three study components (Fig. 1). Component 1 documented the GP process qualitatively, including how it was intended to take place and how it played out in practice. Component 2’s focus was on understanding the effects of GP quantitatively, both proximal effects on health systems and service delivery (e.g., health worker turnover, service discontinuation) as well as distal effects on service coverage through data from DHIS2 health monitoring information systems. Component 3, focused at the facility level, was explanatory, using the experiences of government officials, facility staff and patients to qualitatively understand the effects observed and capture adaptations over time. Components 1 and 3 focused on CS counties over two time points to capture changes over time, whereas Component 2 focused one time point and included three non-CS counties as comparisons. The tools for Components 2 and 3 were pilot-tested in four non-study site facilities, and revised where necessary. Each component is explained in further detail below.

Results from the overall study were presented in a stakeholder workshop held in Nairobi in May 2018 to validate results. All interview respondents at national and county level were invited to participate (no FGD participants were invited). Thirteen individuals attended the workshop, including representatives from USG, IPs, civil society, and four out of six CS study counties.

### Component 1

We conducted a document review and interviews at the national-level to describe the nature of the GP strategy and plans for its implementation. Two rounds of semi-structured interviews (May 2017 and November 2017) with 23 purposively selected key informants from USG agencies, IPs, and relevant units from GOK yielded 22 interviews (Table 1). Five respondents were interviewed in both rounds: 2 from USG, 2 from IPs, 1 from civil society. USG suspended its relations with the Ministry of Health (MOH) in May 2017^2^ [28] during our data collection and at the end of our study had not resumed, limiting our ability to interview government officials.

**Table 1:** Number of Component 1 Interviewees by Respondent Type

Respondent type	Round 1 (Number of interviews)	Round 2 (Number of interviews)
Government of Kenya	1	1
USG agencies	4	4
Implementing partners	3	4
Civil society organizations	4	2
Total	12	11

Interviews lasted between 60 and 90 min, and were conducted in private spaces either in the respondent’s office or off-site to ensure confidentiality. The study used an interview guide that reflected all the key themes (see Supplementary files for interview guides).

Interviews were conducted by DR and MQ who both have graduate degrees in public health and extensive qualitative research experience. CM, OE and the interviewers reached out to respondents in advance via phone or email to explain the study and invite them to participate. During the first round of data collection, the study team was not familiar with respondents, so interviewers spent time early on during the site visit to establish rapport with respondents and explain the study objectives.

#### Analysis

The semi-structured interviews were audiotaped, transcribed, and systematically coded using a thematic analysis approach with Atlas.ti coding software (Atlas.ti 8). Key domains of interest were identified in advance, and included how and when decisions around GP were made; how communications around GP took place across actors and health system levels; and how GP was implemented. DR and other parent study team members piloted the codebook with 1–2 interviews per round, and subsequently DR coded all of the data.

### Component 2

**Fig. 2:**
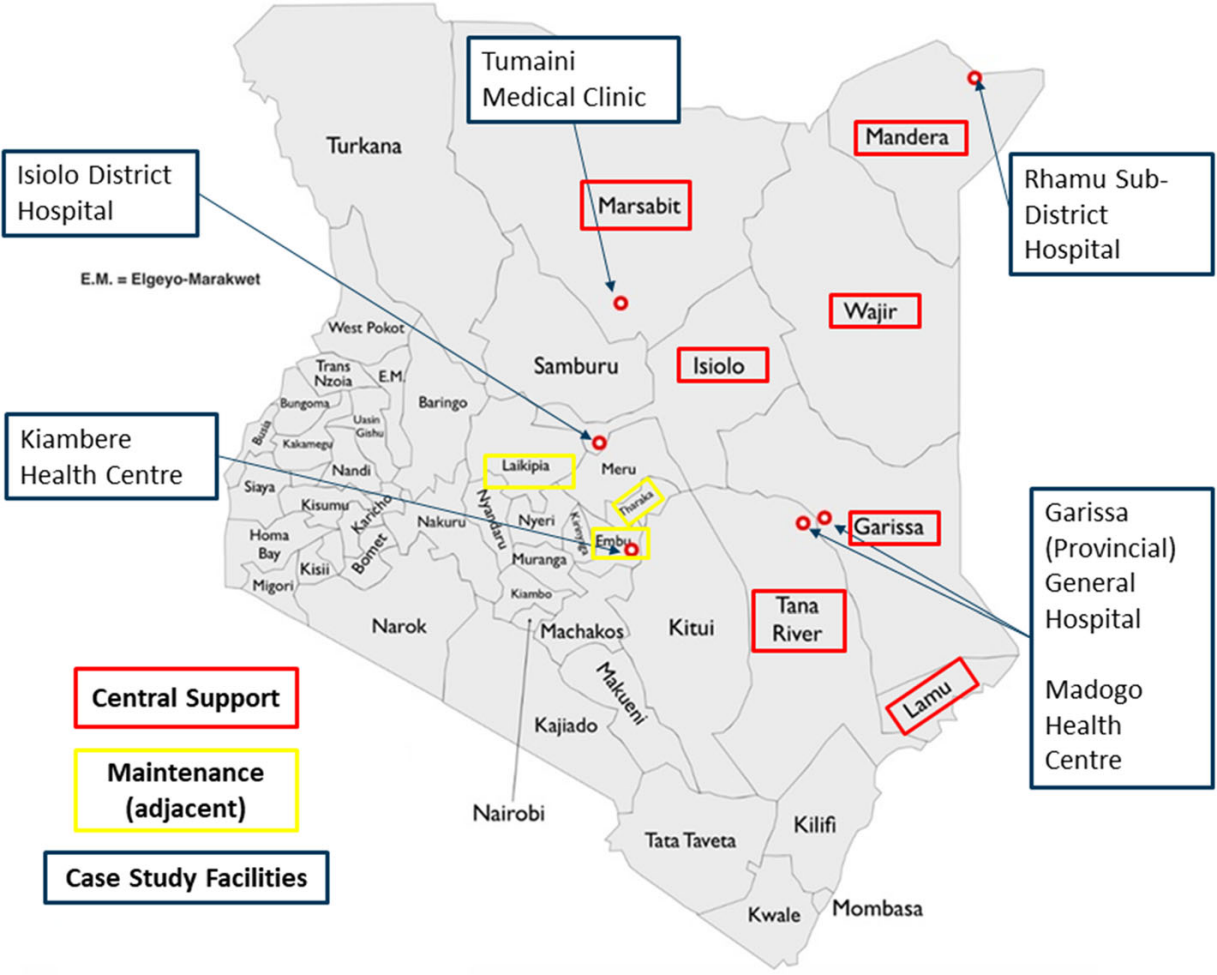
Facility survey counties and case study sites in Kenya, adapted with permission from GeoCurrents [29]

We conducted a survey of 230 facilities as well as an analysis of service delivery data from Kenya’s DHIS2 system. The facility survey was fielded between May and June 2017. We sampled from facilities in all CS counties and three adjacent counties that were classified as “Maintenance” (Embu, Laikipia, Tharaka Nithi) serving as comparisons (Fig. 2). Due to security concerns near the border with Somalia, we excluded areas identified as insecure, all of which were in CS counties, resulting in 64 facilities out of 404 possible CS facilities being excluded from sampling.

We used health facility levels in sampling. In the Kenyan health system, level-2 facilities are health dispensaries staffed primarily by nurses; level-3 facilities are health centers staffed by nurses, clinical officers, and lab and pharmacy technicians [30]; level-4 district hospitals are the first level of tertiary care; and level-5 facilities are referral hospitals for the former provinces.

Three level-5 facilities (Garissa Provincial Hospital, Embu Provincial Hospital, Isiolo Provincial Hospital) were purposively selected because they had the highest level of care in this region. The sampling frame of the remaining 560 facilities was divided into 33 clusters (18 CS & 15 Maintenance). Stratified random sampling was used to select 18 clusters (12 CS, 6 Maintenance). Within selected clusters, all facilities that were level-4 and all level-2 and level-3 facilities reporting more than 10 patients on antiretroviral therapy (ART) in 2015 were selected. We then selected a random sample of 70% of the remaining facilities within the cluster for a total sample of 230 (for more information on site selection see Supplementary File).

Among the 230 facilities surveyed, 37 reported that they never received support from PEPFAR and thus were excluded from further analysis. Of the remaining 193 facilities, 136 reported transitioning to CS support and 57 reported maintaining PEPFAR support (Table 2). Among CS facilities, 83.1% were located in CS counties compared to 21.1% in Maintenance counties. Despite oversampling, most facilities in the unweighted sample were level-2 facilities (64%). The majority of facilities were also government-owned (84%).

**Table 2:** Facility survey descriptive statistics

	Unweighted Descriptive Statistics		
	Central Support	Maintenance	Total
N	136	57	193
CS County	113 (83.1%)	12 (21.1%)	125 (67.8%)
Facility Level			
*2 (Dispensary)*	82	40	122 (64%)
*3 (Health Center)*	40	11	51 (27%)
*4 (Hospital)*	11	5	16 (8%)
*5 (Referral Hospital)*	3	1	4 (2%)
Ownership			
*Public*	117	45	162 (84%)
*Private Not for Profit*	10	3	13 (6%)
*Private for Profit*	9	9	18 (10%)

Surveys were conducted using a standardized instrument specifically developed for this study that assessed past and current support for and status of HIV and MNCH care, laboratory, commodities, finances, and human resources at each facility (see Supplementary Files for survey). Questions focused on how the facility had been prepared for transition; and elicited respondent perspectives on both the impact of transition on services and facility operations and shifts that had taken place since the transition date. Despite efforts to track government and donor financing data for HIV and non-HIV services, these were not available at facility level and are not reported on further. The survey took 60 to 90 min to administer, and respondents were compensated KES1000 (USD $9.5) for their time.

In smaller facilities, surveys were conducted with facility in-charges or their acting replacements. In larger facilities, multiple respondents (e.g., in-charge, lab manager) contributed to the survey. The majority of primary respondents were nursing officers (69%), with a minority of clinical officers (15%). Other cadres of respondents included HIV Testing and Counseling (HTC) counsellors (3%), community health workers (3%), and medical officers (2%). A third of respondents had worked at the facilities for longer than 5 years and 34% for 2–5 years. Only 18% of respondents had worked at the facilities for less than 1 year. Whenever primary respondents lacked information on a particular topic, secondary respondents knowledgeable about the topic were sought from within the health facility.

In the final part of the survey, the enumerators randomly selected 1–3 facility workers from a complete listing of workers providing HIV services, and administered individual questionnaires asking about non-salary incentives, changes in work time-allocation since GP, job satisfaction, etc. The secondary questionnaire was administered to workers in private, away from other staff. A total of 351 individual interview responses were collected across Maintenance and CS facilities.

#### Health service delivery data

We extracted DHIS2 data on HIV and select non-HIV health services for PEPFAR facilities in the 10 survey counties covering October 2013 to December 2017 (Table 3). Following extraction, we merged DHIS2 data to lists of PEPFAR-supported facilities.

**Table 3:** Tabulation of facilities with available data for HIV and non-HIV indicators of interest

Indicator	N Transition (Out of 136)	N Maintenance (Out of 57)	N Total (Out of 193)
Current Patients on ART	59	31	90
Cohort Retention (12 months) on First-line ART	38	30	68
HIV Testing & Counseling (HTC)	108	53	161
New Patients on ART	108	53	161
HIV Testing in antenatal care (ANC)	118	53	171
Syphilis Testing in ANC	118	53	171
Facility-based Deliveries	118	54	172
Total ANC Visits	118	54	172
Fully Immunized Children < 1 year	118	52	170
Outpatient Department Visits	119	54	173

We initially expected GP to occur sometime in mid-2016. We defined facilities that reported to DHIS2 at least two times pre-GP (October 2013 to June 2016) and at least two times post-GP (July 2016 to December 2017) as having enough data for analysis. We removed highly out-of-range data values that could bias analysis, by taking the average of each field by facility and identifying large outlier values relative to the facility average. We also checked the largest 1–5% of cases to look for improbably high values given the facility size. Less than 1% of cases were excluded for most of the above indicators.

Importantly, two widespread clinician strikes over salaries and work conditions took place in the immediate post-transition period. Doctors were on strike from December 2016 through March 2017, and nurses from June to November 2017, which we address in the interpretation of our results.

### Component 3

Longitudinal case studies in six purposively-selected facilities (5 CS, 1 Maintenance) were used to examine how prepared facilities were for GP, how the GP affected the health system and service delivery, anticipated and unanticipated consequences, and how different actors, including patients, experienced GP. For each facility, we conducted interviews with health facility managers, county health officials, IPs, and FGDs with patients to gain a holistic and in-depth investigation of the transition process over time, including adaptation to transition. The study used respondent-specific interview guides for each respondent type, including patient FGDs, which reflected all the key themes with specific considerations for the respondents’ role (see Supplementary files for guides.)

Case study selection considered the level of the health facility, GP category, and patient volume (Table 4). Case study facilities were selected from the facility survey sample. Since there are no unique patient identifiers in the health monitoring information system (HMIS), we were unable to track care seeking behavior for affected patients. Instead, we sought to understand the consequences of GP on service users through sex-disaggregated FGDs with patients.

**Table 4:** Longitudinal case study sample

Facility	County	Facility investment category	Facility level	# of patients on ART in 2016^a^	Facility ownership
Garissa Provincial General Hospital	Garissa	Central support	5	606	Public
Isiolo District Hospital	Isiolo	Central support	4	1338	Public
Rhamu Sub-district Hospital	Mandera	Central support	4	16	Public
Tumaini Medical Clinic	Marsabit	Central support	2	368	Private not-for-profit
Madogo Health Centre	Tana River	Central support	3	58	Public
Kiambere Health Centre	Embu	Maintenance	3	31	Public

^a^ ART data correspond to March 2016

For each case study facility, we conducted two rounds of interviews and FGDs in May and November 2017. We conducted a total of 36 semi-structured interviews (16 in Round 1 and 20 in Round 2, with some overlap) and 22 FGDs (11 per round^3^). Interviews lasted between 30 and 75 min, and FGDs lasted 60 to 90 min. All were conducted in private spaces to ensure confidentiality (e.g. office, clinic tent.) Respondents were compensated for their time (KES 500 (USD $ ~ 4.7) per respondent), and FGD participants were also offered refreshments.

Interviews were conducted by trained research assistants (RAs) hired by Ipsos-Kenya (local data collection agency) and supervised by CM and EO. RAs were deployed in mixed-gender teams to each case study location to ensure that respondents could be interviewed by someone of their same gender, where appropriate and necessary. RAs were experienced qualitative researchers, and they received study-specific training about the study objectives, sample, tools, and overall research ethics. For interview respondents, RA teams reached out in advance via phone or email to explain the study and invite them to participate. FGD respondents were recruited via each facility’s patient support group mobilizer to provide an initial invitation. The study team was not familiar with the study sites in advance of data collection, so the RA teams spent time early on during the site visit to establish rapport with respondents and explain the study objectives.

#### Analysis

The semi-structured interviews and FGDs were audiotaped, transcribed, translated as needed, and systematically coded with Atlas.ti (Atlas.ti 8) using a thematic analysis approach. Domains of interest identified in advance include changes to service delivery post-transition, shifts in care seeking among the patient population, relationships among different stakeholders and communication between them, positive and negative consequences emerging from transition, and strategies arising to address them. EEA, MQ, DR, and other parent study team members piloted the codebook with 1–2 interviews per round, and subsequently EEA coded all of the data.

Each case study facility was analyzed and summarized as a whole over time, followed by cross-case comparisons to identify common experiences across facilities as well as outliers. Results from the case studies were compared with quantitative results to illuminate findings and develop a holistic view of the GP.

## Results

Nearly all surveyed facilities reported transitioning during the latter half of 2016, which was consistent with interview data. Transitions reported before 2016 were more common in CS counties than in the adjacent counties.^4^

### GP process

Almost all national and county level interviewees were aware of GP at the time of their interview and understood that GP was prioritizing investments by burden of disease. There were more mixed reports among facility respondents and very few patients were aware of GP. Respondents recognized that GP aligned with GOK’s own prioritization strategy; however, county, facility, and civil society organization (CSO) respondents raised doubts about the accuracy of the data used to develop the GP plan and criticized GP because it contributed to the further marginalization of counties that were already relatively neglected.

The main criticism of the GP across stakeholder groups interviewed was the short timeframe from announcement to implementation (Table 5). The initial announcement for GP was made as part of USG’s 2015 Country Operating Plan (COP) process in late 2015 but this triggered a protracted negotiation period with GOK on the specifics of GP, including a letter from the Council of Governors which represents county governments, especially because the proposed timeline did not coincide with the Kenyan fiscal year, severely limiting the government’s ability to replace the lost support. High-level negotiations took place past mid-2016 and resulted in several changes:

- Shift from facility-based to county-based allocations for investment categories,
- Extension of the transition deadline from December 2015 to September 2016,
- Retaining services for orphans and vulnerable children (OVC) for a longer period, and
- Agreement on a package of support from IPs targeted at the county level (i.e., above-site support) including support for commodities, laboratory networks, data review, and planning.

**Table 5:**
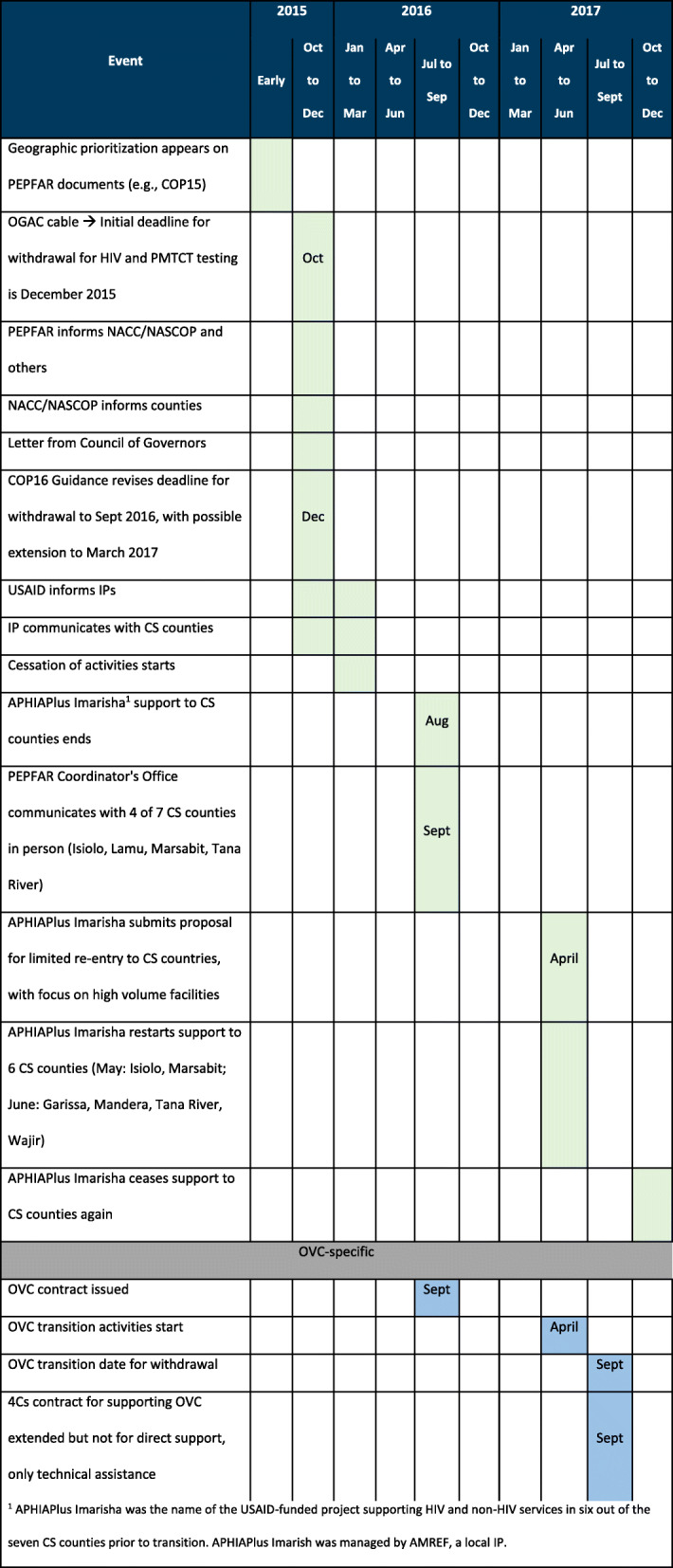
GP Timeline

^a^ APHIAPlus Imarisha was the name of the USAID-funded project supporting HIV and non-HIV services in six out of the seven CS counties prior to transition. APHIAPlus Imarish was managed by AMREF, a local IP

These negotiations, which addressed the very core of which facilities were to be transitioned, resulted in considerable delays to the original implementation timeline.

According to interviewees, USG informed IPs about GP directly, making them responsible for informing counties of the upcoming shifts without backing from PEPFAR or the national government, although that had not been the intention.

*“Particularly for Imarisha [project name] we said ‘look, we are going to try to not hang you out to dry with the counties, and say now you have to go tell the counties that your donor just decided to back out’, we would do it with them. But I think in the near term, ultimately, it did fall back on AMREF [IP] to have those preliminary conversations in the absence of anyone from the US government.” – USG (D4)*

IPs expected counties to inform facilities but that did not happen consistently, and some facilities were unaware of transition until support ceased. Only 33 of surveyed facilities (24%) received communication about GP and of those, 14 were informed after transition took place. It was not until September 2016 that the PEPFAR Coordinator’s Office was able to reach out to counties directly and advise them about GP, but by that point IPs had already ceased support.

#### Planning and preparation

There was widespread agreement across interviewees that planning for the GP was lacking. PEPFAR’s focus on GP planning coincided with COP planning but the execution and roll-out of the GP itself was not formally planned. In fact, no written plan for carrying out GP was developed. Consequently, very little preparation and support was in place for implementing GP, and IPs were left to lead the implementation on their own.

*“Now, that’s where I think the weakness was, was that there was not so much time in terms of planning … We didn’t give like a lag of like two years’ time to implement. It was there and then. That was the biggest criticism that we received … from government.” – IP (D8)*

Partially as a consequence of the negotiations, it was agreed to approach the OVC program differently. USAID issued a separate 1-year contract to conduct assessments and prepare a structured transition process for six out of the seven CS counties (excluding Lamu). Additional support and resources were targeted at raising awareness about impending transition, developing linkages between actors, and building capacity to manage OVC services. Although the timeline was tight, respondents reported the project accomplished most of its goals.

#### Post-transition support

The package of above-site support that had been agreed during negotiations was not delivered as planned due to limitations on the IP’s funding and staff capacity.^5^ Both USG and IP respondents recognized that they had not realized the level of effort necessary to provide above-site services, especially given the remoteness of the CS counties, and acknowledged that they adjusted their strategies over time.

*“Well, the fact that we were initially told we should hand over all these services to the counties. And then suddenly you thought, ‘oh, excuse me guys, you guys have to go back. Things are not looking good and there are no additional resources, work within the resources that you still have.’ That was tough … we had to shift some of our staff in from the aggressive scale-up counties [the IP was also supporting] and bring them here [to CS counties] … And given the geography and the distances we had to cover, that was not easy.” – IP (D25)*

By April 2017, data reported by the IP from CS counties^6^ suggested that HIV services were declining so the IP returned to eight high-volume facilities in six CS counties with USAID approval. The return, locally referred to as a “rescue package”, started in May–June 2017 and lasted until the end of 2017 when the IP’s contract ended. The IP provided much needed office supplies, supplementary lay counselors for testing, laboratory networking, data bundles for information upload, phone cards for defaulter tracing, etc.; however, it was expected that improvements would drop off once the IP left again.

“*So, the fact that APHIAPlus [the IP] came in with a rescue package, barely one year after abrupt cessation of program support, it is an indication that the cessation of support was not planned, it was not structured, and there was no proper exit strategy.” - County HIV official, CS County*

In terms of post-transition support from government agencies, respondents suggest financial analyses and technical assistance were planned but there were inconsistent reports from interviewees about whether it happened. Our inability to conduct follow-on interviews with national government stakeholders limit our ability to describe this further.

About half of CS facility in-charges surveyed reported that the county government was providing support (51%) and/or engaging more in facility operations (54%). Interviewees indicated that GP had forced county officials to become more familiar with and aware of their HIV profile and services, but the low HIV burden in CS counties resulted in low priority. Case study respondents described getting counties to prioritize HIV support as a main challenge.

*“Some counties … is easier simply because of the HIV burden which is visible. If you go to the counties around the Lake Victoria, county leadership would understand that this is a real problem but some counties around the Mount Kenya and also Northeastern Kenya they think they have other priorities.” – CSO representative (D5)*

### Service delivery effects

#### Discontinuation of services

Transition did not impact HIV services on offer, with two important exceptions. Eight CS facilities (10% of the CS facilities providing ART for patients before transition) discontinued providing ART compared to none of the Maintenance facilities surveyed. Most facilities discontinuing ART were level-2 facilities in remote areas. Transition from PEPFAR was the most commonly cited reason for discontinuation of ART services for patients. There were few discontinuations of other services (e.g. PMTCT, immunizations, nutrition) with no significant differences among facility types. However, discontinuation of outreach, including patient identification (outreach testing) and activities for adherence, occurred in 39% CS and 36% Maintenance facilities surveyed. This effect might be more closely associated with an earlier PEPFAR shift to focus on facility-based services (i.e., Technical Pivot) than on GP.

#### Access and quality of services

CS facility in-charges were significantly more likely to report worsening access and quality of HIV services since transition compared to Maintenance (Table 6); perceptions for MNCH services worsened but were not significantly different among facility types. In-charges also identified declines in access for key populations and the poor, which might be linked to declining outreach services. Importantly, impacts were greater in larger facilities (e.g., level 3 or higher).

**Table 6:** In-charge reported access and quality of services

	Access to and Quality of Services	CS (*N* = 136)	Maintenance (*N* = 57)
		Reported worsening	
**HIV**	Average patient access to HIV services*	35.3%	12.5%
	Poorest patient access	41.9%	25.9%
	Key population access*	37.1%	19.6%
	Quality of HIV Care*	27.9%	5.4%
**MNCH**	Average patient access to MNCH services	19.3%	10.9%
	Poorest patient access to MNCH	22.9%	20.0%
	Quality of MNCH Care	12.6%	7.3%

*Significant at *p* ≤ 0.03

#### Service volume

Data on service volume came exclusively from DHIS2. Among HIV services reported to DHIS2, we prioritized three that were available fairly consistently over time: number of HTC performed, number current patients on ART, and 12-month cohort retention on first-line ART for patients in HIV care. The clinician strikes in 2016/2017 affected levels of services provided and appear to have impacted Maintenance facilities more than on CS. In addition, the IP’s “rescue package” included hiring 11 HTC counselors at high-volume facilities during the nurses’ strike.

**Fig. 3:**
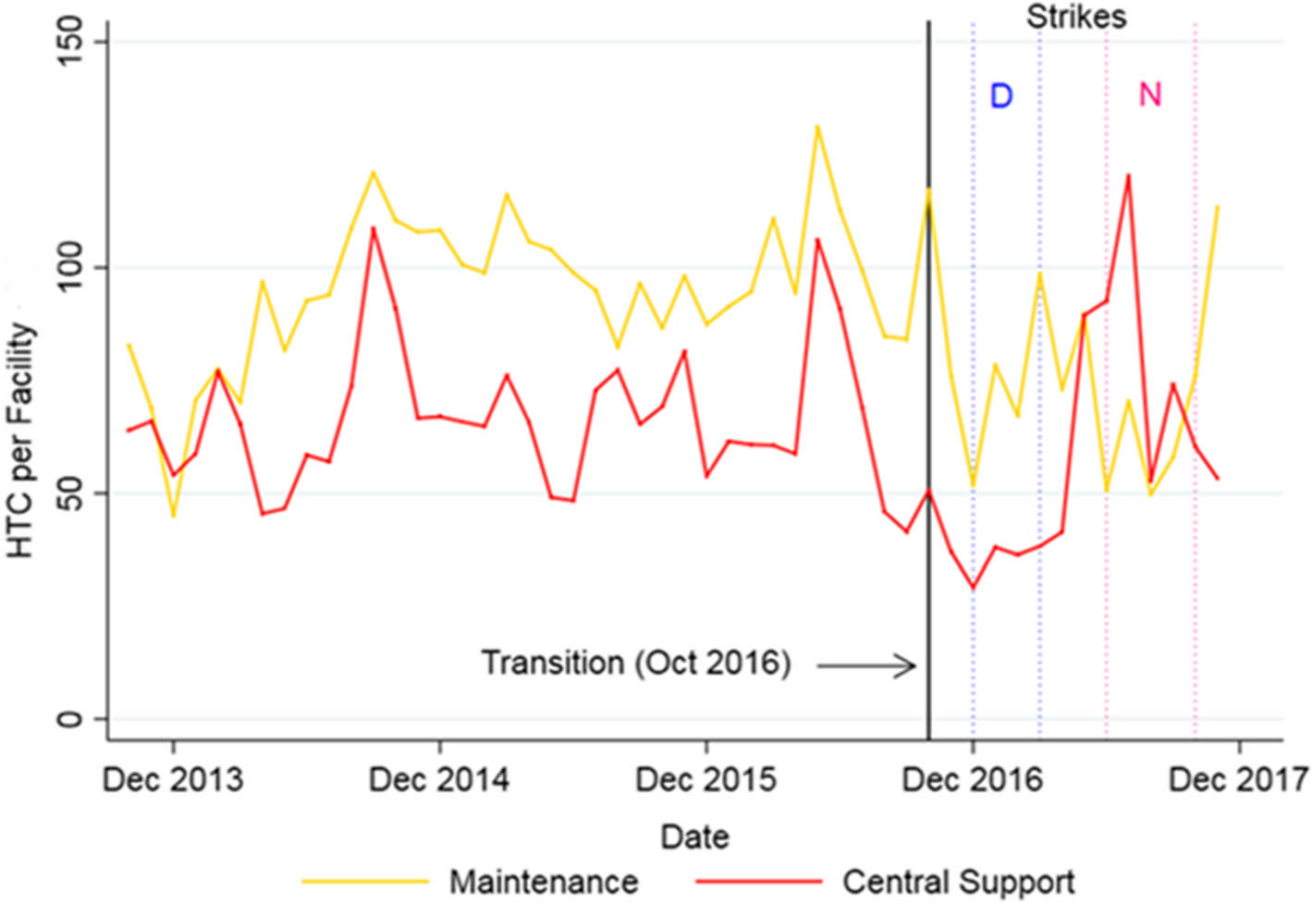
Trends in number of HIV Testing and Counseling (HTC) per facility per month, 2013–2017. Note: “D” indicates the Doctor’s Strike (Dec 2016 – March 2017) and “N” the Nurses’ Strike (June – Nov 2017)

Overall, HTC declined in both CS and Maintenance facilities in the months following transition. There was a surge in CS that appears to coincide with the rescue package, while in Maintenance facilities HTC rebounded towards the end of 2017 (Fig. 3).

**Fig. 4:**
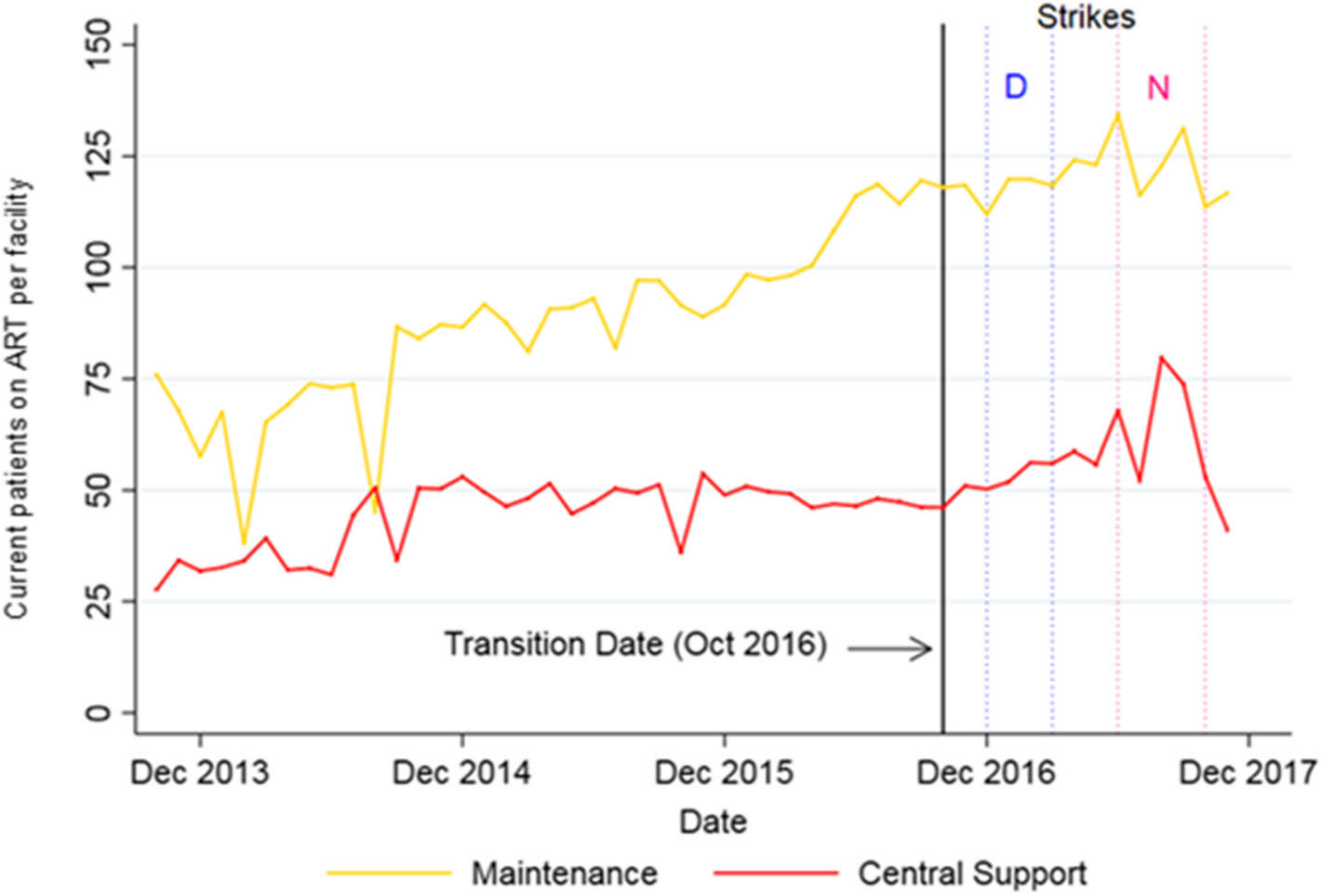
Trends in number of current patients on ART per facility per month, 2013–2017. Note: “D” indicates the Doctor’s Strike (Dec 2016 – March 2017) and “N” the Nurses’ Strike (June – Nov 2017)

For the number of current patients on ART, numbers in Maintenance facilities plateaued around the time of transition, but increased modestly in CS then fell off in late 2017 (Fig. 4).

**Fig. 5:**
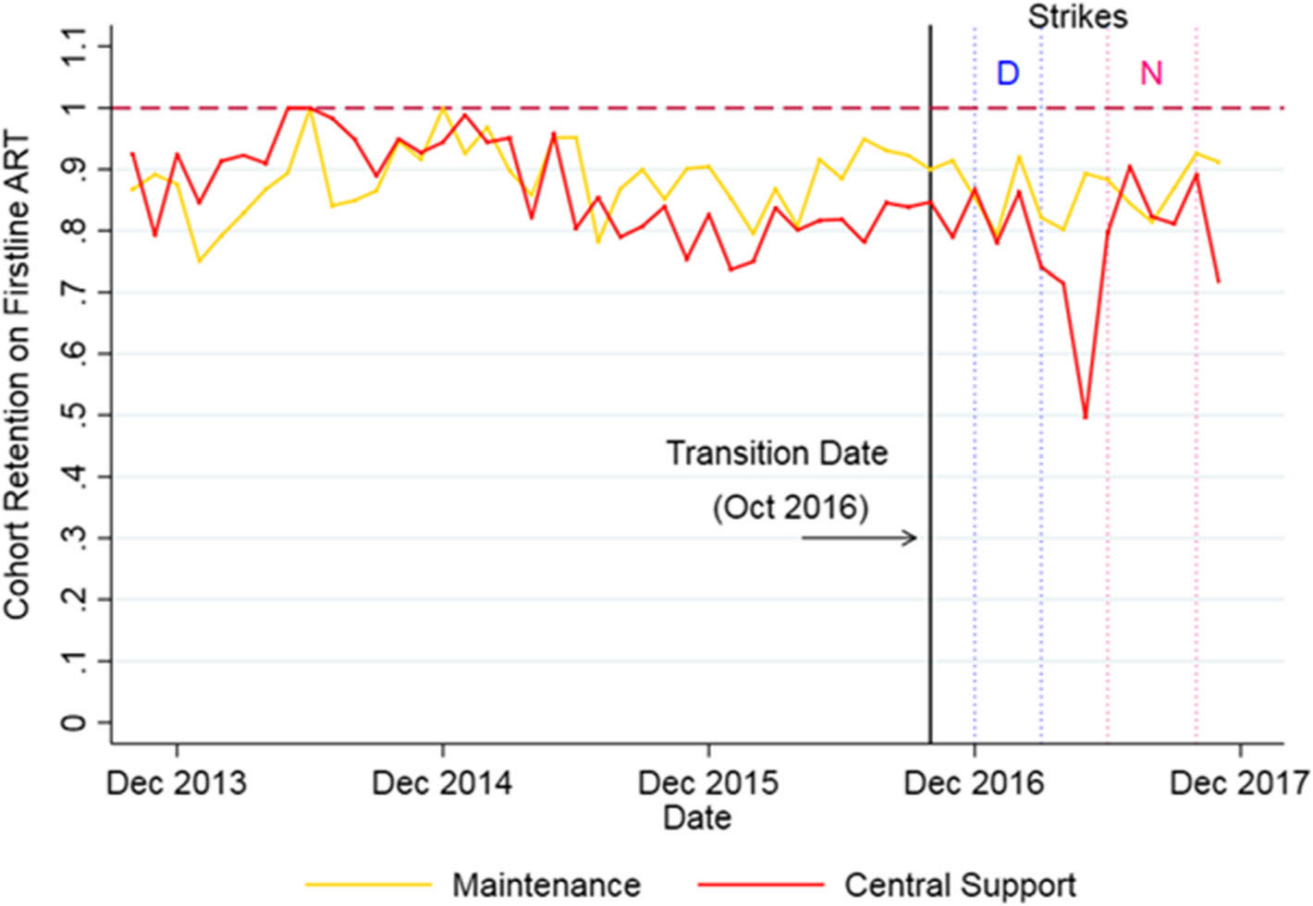
Trends in 12-month cohort retention of patients on first-line ART per month for all facilities, 2013–2017. Note: “D” indicates the Doctor’s Strike (Dec 2016 – March 2017) and “N” the Nurses’ Strike (June – Nov 2017)

For cohort retention, we were only able to include data from 68 facilities (compared to 90 facilities for current patients on ART) with 70% of observations missing. The sharp drop in cohort retention in May 2017 coincides with a lower reporting by CS facilities and might be noise as the decline did not appear to have been sustained (Fig. 5).

**Fig. 6:**
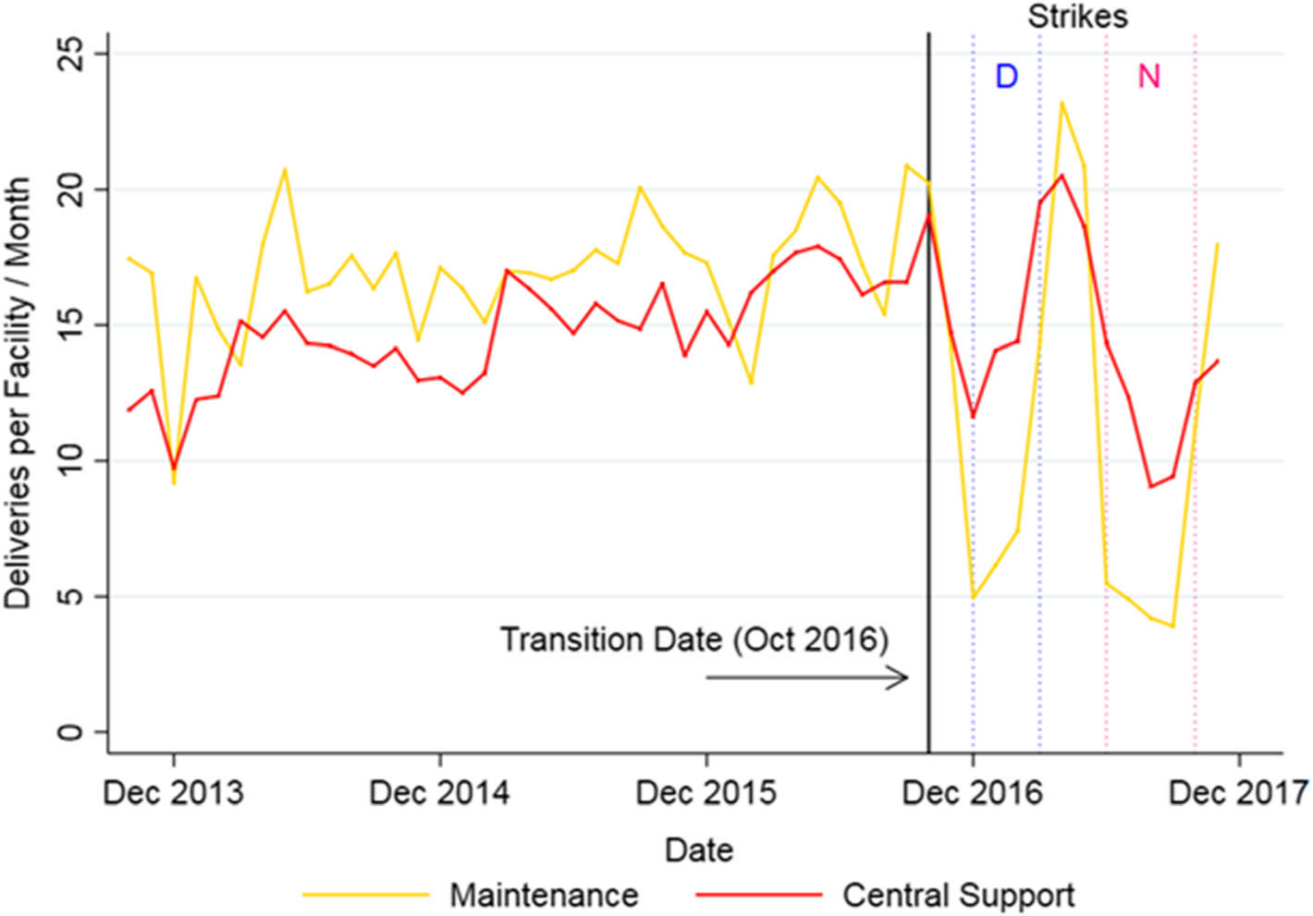
Trends in number of facility deliveries per facility per month, 2013–2017. Note: “D” indicates the Doctor’s Strike (Dec 2016 – March 2017) and “N” the Nurses’ Strike (June – Nov 2017)

For non-HIV services we assessed, Fig. 6 provides an example of the apparent impact of clinician strikes on facility deliveries in both CS and Maintenance facilities.

### Health systems effects

The evaluation explored effects on all health systems building blocks but the main effects were seen around health workforce and laboratory services.

#### Health workforce

Effects on health workforce were reported around time allocation, training, staff turnover, financial incentives, and job satisfaction. Substantial proportions of CS in-charges report that since GP staff spend more time on MNCH (50.7% of facilities) and less time on HIV services (36.5% of facilities), with facilities Level 3 and higher significantly more likely to report declines in time spent on HIV care. In-charges in both types of facilities reported modest declines in HIV and MNCH supervision but were not significantly different.

Seventy-three percent CS facility in-charges reported inability to attend trainings as a result of transition. Almost two-thirds of CS and Maintenance staff also reported declines in time spent in training (65.4 and 59.8%, respectively), though the difference was not significant between them. National respondents suggested the loss of training support might be most acutely felt because although county officials could join ongoing trainings, often there was no funding to attend.

Interviewees reported that formal staff positions previously supported by IPs had been shifted earlier to be hired through county systems in order to support retention. 11.7% of CS facilities surveyed reported terminating staff versus 1.3% of Maintenance facilities (*p* = 0.004), however these appeared largely to be contractual workers such as lay health workers who were not absorbed by government contracts. CS facilities reported higher proportion of vacant posts (25.9%) compared to Maintenance (8.7%) (*p* < 0.001). Case study respondents reported that the private, not-for-profit (PNFP) facility had a massive drop of patients, in part due to loss of staff. Although county officials were initially not willing to replace these positions, the subsequent loss of patients prompted them to post two providers to the facility to address the issue.

The proportion of HIV workers receiving salaries from the IP as well as allowances for outreach declined slightly in both CS and Maintenance facilities and were not significantly different. However, 95% of CS workers that reported receiving bonus/top-ups reported that these declined compared to 40% of Maintenance workers (*p* = 0.003). Case study respondents indicated that outreach services were difficult for counties to replace, undermining facilities’ ability to trace those lost to care.

*“Right now, nobody is there to support these staff to go to look for those mothers when their children turns positive, so they end up missing.”* - *Health worker, Isiolo County (D55)*

Despite the loss of non-salary support and opportunities for training, workers’ job satisfaction did not differ significantly between CS and Maintenance facilities.

#### Laboratory services

Forty-seven percent of CS in-charges reported laboratory stock-outs as a consequence of the transition, and 52% reported lack of access to specialized testing; however, at the time of the survey there were no significant differences between CS and Maintenance facilities’ ability to provide most laboratory tests. Case study interviewees, including patients, reported HIV-associated testing (e.g., hematology, blood chemistry) stopped or incurred fees for patients because these were no longer subsidized post transition.

Viral load testing was significantly affected with CS facilities reporting higher disruption of testing compared to Maintenance facilities (35% vs 5%, *p* = 0.010). CS facilities were more likely to lack support for lab specimen transport (80.9% v. 53.7%, *p* < 0.001) and for collecting lab results (77.8% v. 55.9%, *p* = 0.001) than Maintenance facilities. While CS facilities should have relied on a regional laboratory hub, interview data highlighted that laboratory services suffered from lack of salary support for hub riders transporting samples to labs and lack of funding to cover data bundles for health staff to download lab results, which resulted in delays delivering results to patients. Finally, these logistical problems were exacerbated by the simultaneous roll out of “test and treat” policies leading to high workloads for laboratories.

*For services such as early infant diagnosis, fast turnaround time is key to eliminating mother to child transmission. It should be two weeks turnaround time, but takes longer in those [CS] regions – 1 month, 3 months? “It is like passing a death sentence to our innocent newborns” (respondent emotional). – USAID (D26)*

*“They tell us that they [VL tests] have been sent to Nairobi and they are yet to come, I have come for four clinics and yet the results have not been brought”. - Male Patient, Isiolo County (D64)*

By the time of the second round of qualitative data collection, the testing situation had improved—in part due to the “rescue package”—but not completely resolved, and respondents recognized that laboratory quality was suffering due to declines in training on new protocols and regular quality assurance.

#### Commodity supplies

CS facilities were not significantly more likely than Maintenance to report stock-outs of any one of the 12 tracer commodities. In-charges in 43% of CS facilities reported delays in drug and supply orders had taken place due to GP, and at least one case study facility reported swapping/sharing test kits between facilities as a coping mechanism post-transition. Although our survey had a low proportion of private facilities, the PNFP case study facility reported difficulties accessing commodities and/or preferred pricing through KEMSA (the government procurement agency).

ART access for patients was widely acknowledged by all types of respondents to have been unaffected by GP and to remain free of charge; however, patients in three CS case study facilities reported having to pay fees for non-ART HIV drugs, which appeared to improve by our second round of data collection.

#### Health information

Twenty-eight percent of CS in-charges reported delays in HMIS submissions since GP. This might be partly explained by case study data indicating that the IP previously provided technical support, reporting forms, and transportation of forms between levels – all of which was lost post-transition.

### Factors influencing the GP

#### Accountability, transparency, devolution and GP

Accountability and transparency around the GP were emergent themes. CSOs felt that formal agreements between development partners and the government, such as PEPFAR’s Partnership Framework (PF) agreement and Global Fund’s counterpart financing requirements, had created a space for civil society to demand government’s compliance with its commitments. The lack of a formal plan for GP was seen as a problematic for two reasons. CSOs felt that without such a plan it was difficult for them to hold government accountable to stepping up its support for transitioned counties and activities, and it also undermined the MOH’s ability to advocate for more funding.

*“That [PF] was very useful for us because it made the government also commit to certain things … Ever since that framework elapsed, their [GOK] support has been … it’s just charity, there is nothing that compels the government of Kenya to put any support because it was only in that time that we saw the government of Kenya respond … We were tracking that [PF agreement] and reporting and saying ‘You said you would hire nurses at district and constituency level. Where are those … nurses? And you said you will increase health budget allocation and where is it?’” – CSO representative (D5)*

Issues around transparency of decisions and planning for GP were raised by multiple interviewees. In particular, county actors questioned why they were not involved in decisions, or even informed, about GP when it would affect them so acutely. National level officials saw GP as a national/higher level decision that should not affect counties because channels of funding would not be changing in a functional way (e.g., ART purchasing was unchanged) so participation and information were not necessary. However, the support provided by IPs was not functionally replaced, including outreach, and counties were at a loss of how to replace such support on short notice.

Implementation of the GP was also made more complex by the relatively recent devolution of health systems governance in Kenya, and ongoing confusion around roles. Whereas county respondents saw HIV, tuberculosis and malaria as national public health programs that the federal level would support, in reality the national role is limited. Respondents identified a number of functions previously undertaken by IPs, including capacity building and consumer protection (ensuring quality of commodities), where there was lack of clarity about which level of government was responsible.

Interviewees expressed widespread expectation that government actors would step in to support CS counties and replace IP support, but this had not happened or not happened fully. Counties frequently recognized their responsibility but did not have the budget to replace PEPFAR support due to limited resources. Although CS counties were not prepared for GP, some were more willing or able to take on the changes. Counties such as Isiolo and Marsabit were described as being interested in taking on leadership roles in ensuring services continued to be delivered; others, such as Tana River, were much weaker in terms of implementing existing services, and unable to take on responsibility for overall support. Many respondents raised the challenges of getting CS county governments to prioritize HIV in light of many other competing health issues with higher burden and profile, such as MNCH.

#### Outreach’s effect on services

The predominant concern emerging from the case studies was the loss of support for outreach. This was linked to several issues, most notably the loss of outreach workers and expert patients, reduced allowances for transport, and to a lower extent challenges in the lab network. It was noted that losing outreach had affected the ability of facilities to conduct defaulter tracing. Health staff believed this would translate into increases in the number of patients lost to follow-up (although there was no clear evidence of this at the time). Respondents in some CS facilities also suggested that there was a drop off in testing, particularly among hard-to-reach populations such as nomads, and patients who faced high stigma in accessing services. There were also complaints that the loss of dedicated HIV counsellors affected privacy, and concerns that new staff hired by the county was less responsive to patients.

*“This is a community where stigma is very high. So, convincing somebody to undergo HIV test here, it is not an easy thing. So, if you do not conduct outreach, the number clients who are going to come to the VCT to seek for these services are very low.” – County HIV official, CS County (D67)*

*“Since May [2017], many clients were lost to follow up. Even today we cannot be able to trace them. So many people left drugs, even today we don’t know where they are, whether they went to other facilities or whether they died, we cannot give a report about that.” – Health worker, Marsabit County (D73)*

## Discussion

This study of the effects of facility-level transition of HIV programs on services, health systems, and coverage in Kenya present a mixed picture. Although there were no major effects on HIV and non-HIV service delivery to-date, two protracted clinician strikes and the “rescue package” were major confounders. Our survey and qualitative data suggest modest effects on HIV service discontinuation, including a few smaller facilities discontinuing ART for patients and widespread discontinuation of outreach in both CS and Maintenance facilities. CS facility in-charges also reported a failure to improve HIV and MNCH service access and quality which might eventually affect coverage. Notably, most effects were worse among higher-level facilities suggesting that these require more attention when planning transition, perhaps due to their higher initial reliance on support from IPs. Future research should explore effects of GP over a longer time period and document how local actors enact coping mechanisms as they shoulder more responsibility for services.

Positive aspects of GP in Kenya included broad-based agreement among key stakeholders to a prioritized approach, alignment of GP with GOK’s own agenda, and willingness among key stakeholders, especially PEPFAR, to negotiate on details, such as county-based allocations. Alignment between donor and government priorities was also seen as a strength in the FP transition in Mexico [20], underlining broader goals in development assistance. Two serendipitous characteristics that helped GP in Kenya were having one IP managing 6 out of 7 CS counties limiting operational confusion (in contrast to Uganda [27]), and centralized procurement for ART was already well-established, which had proven problematic elsewhere [13, 20].

However, the GP process was marked by short timelines, poor communications and lack of planning, which is consistent with critiques of PEPFAR’s early transition efforts in South Africa [31] where disruptions on ART access were linked, in part, to limited pre-transition planning [32, 33]. Challenges in Kenya were compounded by the recent devolution. Actors consistently underestimated the extent of the impact that transition would have at the local level. National-level decision-makers had insufficient understanding of exactly what support would be lost and what it would require to replace it (e.g., delivering above-site support), which was compounded by the short notice given to county officials. Further, county officials are now facing a balancing act between HIV and other, higher burden issues, and might not prioritize HIV. Concerns about transition in decentralized contexts have been already seen elsewhere [20] and have been flagged by UNAIDS [34]. Further, the lack of a transition plan for GP limited the options for demanding accountability from government, including CSOs.

There were several limitations to this work, including the dynamic context in which transition took place, such as clinician strikes, making it difficult to isolate the effects of GP. Second, study and DHIS2 data reflected a relatively short period of time post-transition, so other impacts or course corrections might become evident in the future. Also, the availability, completeness and consistency of DHIS2 reporting limited our ability to analyze all tracer indicators and might obscure effects from GP. Third, limited access to national GOK respondents following the USG suspension limited our ability to reach saturation, and might skew results and interpretation. During the second round of data collection, we specifically sought out former national GOK respondents in order to inform our understanding of the government’s position on GP. Fourth, few private facilities were available for inclusion but qualitative data from one PNFP suggested they might suffer more than public facilities during transition. Lastly, the perceptions of survey and case study respondents were generally more negative regarding the effects of GP than the DHIS2 suggest, which raises the possibility that respondents were exaggerating their concerns. A longer study period post-transition would strengthen the analyses of effects, and would minimize the impacts of confounders, such as clinician strikes and data quality, on interpreting the results.

Our findings provide additional empirical support to recommendations calling for readiness assessments, planning with reasonable timelines and improved coordination, and participatory and inclusive communications [19, 20, 24, 35]. They also raise larger questions about underlying drivers for donor transitions. Although GP makes sense epidemiologically, and was aligned with GOK’s own position, it resulted in transitioning weaker counties that had been historically under-invested and face many other competing priorities, making them less likely or able to take over the responsibilities that PEPFAR was seeking to transfer. Further, Kenya presents a scenario where GOK, USG, and even Global Fund are all prioritizing their efforts geographically, which leaves transitioned areas with few avenues for support.

Furthermore, the change in HIV programming to target high volume sites and reduce community-based initiatives requires reflection. Losing community-oriented outreach in hard-to-reach areas with high stigma only complicates efforts to reach UNAIDS’ 3rd target on ART adherence. Further, what are the implications for achieving national targets when sub-national efforts are weakened? While concentrating attention on high-yield interventions makes sense to achieve national targets, neglecting remote and marginalized populations with low-prevalence worsens inequities. Worse effects for key populations post-transition were identified in Romania and Serbia after the Global Fund transition [33]; although our study does not address key populations explicitly, it remains a potential concern in CS areas.

## Conclusions

Earlier transitions have seen donors exit a country completely, but within-country transitions are becoming more common. This study highlights both implementation challenges that arise from transition as well as broader, more philosophical questions about the trade-offs that take place as priorities for global, national, and local actors shift. Increasing demands for domestic resource mobilization force local actors to balance priorities between HIV and other issues (including non-health concerns) in ways that may deprioritize HIV and will require greater advocacy by development and civil society actors than has been conducted to-date.

## Supplementary Information

**Additional file 1.**

**Additional file 2.**

**Additional file 3.**

**Additional file 4.**

## Data Availability

The datasets used and/or analyzed during the current study are available from the corresponding author on reasonable request.

## Abbreviations

ART: Anti-retroviral therapy
COP: Country Operating Plan
CS: Central support
CSO: Civil society organization
FGDs: Focus group discussions
GOK: Government of Kenya
GP: Geographic Prioritization
HTC: HIV testing and counseling
IP: Implementing partner
MNCH: Maternal, neonatal and child health
NACC: National AIDS Control Council
NASCOP: National AIDS/STD Control Programme
OVC: Orphans and vulnerable children
PF: Partnership Framework
USG: United States government

## Footnotes

1 Under devolution, shareable central-level funds are redistributed in order that 14 historically marginalized and under-invested counties are cross-subsidized by wealthier counties. Nevertheless, within county allocations between sectors are at county governments’ discretion resulting in great variability in county budgets for health.

2 USG suspended over USD 20 million in assistance to the Kenya MOH over concerns of corruption and poor accounting. Direct funding for HIV treatment and other health services were not suspended.

3 No FGDs with male patients were conducted in Mandera county in either round because no patients were willing to attend.

4 Survey respondents were asked to date the time when they lost PEPFAR support. We believe, but could not confirm, facilities losing support prior to 2016 were unrelated to GP. For example, many facilities reporting early “transition” were located in Mandera county, where security has been an issue for several years prior to GP.

5 In addition to the six CS counties, the IP also retained one Maintenance and one Scale-up county in their portfolio during this period, which is where they focused their resources.

6 It was not clear whether these data came from the IP, the counties directly, or PEPFAR’s data systems but they differ from DHIS2 for this period.
