# Supplementary material for: Effects of transition on HIV and non-HIV services and health systems in Kenya: a mixed methods evaluation of donor transition
Source: BMC Health Serv Res. 2021 May 13;21:457. doi: 10.1186/s12913-021-06451-y (PMC8117613; doi:10.1186/s12913-021-06451-y)
Supplement: Supplementary file 1 — Additional file 1. [file 12913_2021_6451_MOESM1_ESM.docx]

SOAR Kenya Paper Overall Results

Supplementary File

# Component 2 Methods, detailed

The number of clusters and facilities for the facility survey was chosen as a result of 1,000 Monte Carlo simulations [6]. In each simulation, we apply hypothetical scenarios to the data and sample repeatedly to determine in what proportion of simulations we are able to detect the hypothetical “truth” from our sample, i.e. the power. We sought to have 80% power to detect a shift in responses among the CS facilities of 30% of sampled facilities with a 4:1 ratio of Yes-to-No compared to No-to-Yes changes at a 5% Type-I error level. This required a sample of 147 facilities in the seven CS counties. We also sought to achieve a 2:1 ratio of CS to maintenance facilities to allow for comparisons both between CS and maintenance facilities and among CS facilities. Therefore, we added 6 Maintenance county clusters for a total of 18 clusters and 230 facilities.

Lacking data on reasonable expected non-response rates prior to this study, we opted to identify replacement facilities in Kenya rather than to oversample. We selected one or more replacements for each sampled facility from unused facilities in the sample frame. Replacements were drawn first from facilities with the same ownership category (private not for profits (PNFP), private for profit (PFP), or government), level, and cluster. When replacements with these characteristics were unavailable, we selected replacements within one level difference (e.g. level 3 for level 2), similar ownership (private and private not for profit vs. government), or the same level and ownership from nearby clusters. A total of 30 replacements were used^[[1]](#footnote-1)^.

In the analysis, we adjusted for the clustered selection and stratification using the SVY commands in Stata with weights to account for oversampling. Replacements were assigned to the cluster of the facility that they replaced. However, replacement facilities were treated as coming from the strata to which they belonged (e.g. if an ART facility with more than 10 patients was replaced with a non-ART facility, the replacement would be included in the non-ART strata). However, the dataset contains the replacement facility’s covariate data (e.g. level, ownership).

1. During fielding of the survey, the security situation in El Wak, Mandera County was deemed too precarious for data collection, requiring 7 replacements. Three other facilities (one in Lamu, one in Garissa, and one in Marsabit) were also excluded because of local insecurity or access issues. In 14 facilities, there were no staff knowledgeable about the pre-transition situation to interview. Six facilities were either closed permanently, temporarily, or were not open following two visits. [↑](#footnote-ref-1)
