# Supplementary material for: Effects of transition on HIV and non-HIV services and health systems in Kenya: a mixed methods evaluation of donor transition
Source: BMC Health Serv Res. 2021 May 13;21:457. doi: 10.1186/s12913-021-06451-y (PMC8117613; doi:10.1186/s12913-021-06451-y)
Supplement: Supplementary file 2 — Additional file 2. [file 12913_2021_6451_MOESM2_ESM.zip › SOAR_Comp1_IDI Guide 2nd Round_2017 10 10_KenyaR2.docx]

Project SOAR – Documenting the PEPFAR Geographic Prioritization

Semi-Structured Interview Guide – National-level Stakeholders 2^nd^ Round KENYA

(REPEAT RESPONDENTS ONLY)

# Introduction

Thank you for agreeing to meet us.

We are conducting an assessment of PEPFAR’s geographic prioritization process; that is, the process through which counties/districts and sites fall into different investment categories. We are interested in the processes that took place before prioritization to prepare and after prioritization to implement the changes. Our goal is to provide practical information to local and national government, PEPFAR and other partners about how the geographic prioritization process took place and whether it has affected how services are delivered.

As part of the overall evaluation, we are documenting how the prioritization process has unfolded over time. We have been interviewing various stakeholders about this process over the last 6-12 months to understand what decisions were made about the prioritization, how these were communicated, what changes were made to plans as they are implemented, etc. We conducted a first round of interviews earlier this year, and we have returned to understand how things have changed since then.

| Name of Organization |  |
| --- | --- |
| Your name |  |
| Designation |  |
| Work Area |  |
| Postal address |  |
| Telephone |  |
| E-mail address |  |

**OBTAIN INFORMED CONSENT**

*NOTE TO INTERVIEWER: This is a guide to the interview. You should cover* ***all the main numbered questions*** *in this interview form. You should use the probes selectively, according to the type of knowledge that the respondent conveys, and what you have already found out from documents and other interviews.*

# Interview Questions

## Geographic Prioritization Status and Changes

First, I’m going to start by asking general questions about PEPFAR’s geographic prioritization.

*INTERVIEWER: If respondent was interviewed before and position has not changed, skip Q1. If respondent’s position has changed, ask Q1.*

1. Can you tell me a little about your current role?
2. This is the timeline for we have developed thus far about how the Geographic Prioritization was communicated and to whom. How well does this reflect your experience?
   1. What changes would you propose to this timeline?
      1. When were IPs informed about Geographic Prioritization? Who informed them?
      2. Do you have access to the letters sent by various GOK actors? What did the letters say? [ASK if copy of letter can be shared with project team members.]
   2. When did Afya Pwani wind down support to Lamu?
3. Have there been any changes to the Geographic Prioritization policy or process since May 2017?
   1. When were these decisions made?
   2. How and why where these changes made?
   3. Who explained the process to you and your colleagues?
   4. Have any counties or sites changed investment categories since May 2017? If so, why?

## Support for Services

Now I’m going to ask you about how support for services has changed since PEPFAR’s geographic prioritization.

1. How has the support that counties and sites received for HIV services changed since Geographic Prioritization began? E.g. staff hiring and salaries, commodities, training, funding, support for reporting, patient incentives, etc.
   1. How has this varied by service type? E.g. testing, treatment, prevention, outreach, lab testing
   2. Any changes to how support is provided?
   3. Were all supported areas shifted at the same time?
   4. How has this varied by investment category? E.g. Saturation, sustained, central support
2. How has the support that counties and sites received for non-HIV services, such as maternal and child health, changed since Geographic Prioritization began? E.g. staff hiring and salaries, commodities, training, funding, support for reporting, patient incentives, etc.
   1. How has this varied by service type? E.g. antenatal care, family planning, immunization, malaria
   2. Any changes to how support is provided?
   3. Were all supported areas shifted at the same time?
   4. How has this varied by investment category? E.g. Saturation, sustained, central support
3. For those areas that are no longer supported by PEPFAR, how are these supported now?
   1. Which organization is supporting them?
   2. How was this decided?
   3. How well is it working?
   4. How do you expect this support to look like in the near future? In the mid-term?
4. We understand new funding mechanisms were put in place for supporting HIV services earlier this year. What is different about these mechanisms than the APHIA programs?

*PROBE*: contract type, period of performance, implementing partners.

- 1. How similar or different are each program’s catchment areas than the catchment areas under APHIA? For example, does [NEW IP NAME] work with the same counties as APHIA [OLD IP NAME]?
  2. Why were these catchment areas [kept the same / changed]?
     1. How was it related to Geographic Prioritization and allocations between investment categories?
  3. Is there any support to Central Support counties at all? If so, what?

1. How did the process of switching between implementing partners and/or funding mechanisms go?
   1. Where there any breaks in support to counties? If so, what happened?
2. How are non-HIV services that used to be offered through the APHIAs provided now?
   1. Are non-HIV services also prioritized geographically?
      1. If so, which services are prioritized and why?
   2. How does this support vary by investment category?
      1. Is there any support to Central Support counties at all? If so, what?

## Effects of Geographic Prioritization

Now I would like to ask you a series of questions about the effects of PEPFAR’s geographic prioritization thus far.

1. What effect has the geographic prioritization had on HIV service delivery?
   1. How has the geographic prioritization affected service coverage?
   2. How has the geographic prioritization affected:
      1. Staff turnover?
      2. Staff motivation or performance?
      3. Availability of commodities?
      4. Reporting of data?
   3. How has the geographic prioritization affected service quality and patient satisfaction?
   4. Do you think there have been different effects from the geographic prioritization for public and private health care providers?
2. What effect has the geographic prioritization had on non-HIV service delivery, such as maternal and child health services?
   1. How has the PEPFAR geographic prioritization affected service coverage?
   2. How has the PEPFAR geographic prioritization affected:
      1. Staff turnover?
      2. Staff motivation or performance?
      3. Availability of commodities?
      4. Reporting of data?
   3. How has the PEPFAR geographic prioritization affected service quality and patient satisfaction?
   4. Do you think there have been different effects from the PEPFAR geographic prioritization for public and private health care providers?
   5. What has been the effect of prioritization of RMNCH support that started in October 2016?
      1. How does it differ from Geographic Prioritization under PEPFAR?
3. Since May 2017, how have different organizations changed the way they operated as a result of PEPFAR’s geographic prioritization?
   1. Examples:
      1. Counties or facilities: changes to reporting, staffing, support for service delivery, etc.
      2. National government, like NACC and NASCOP: changes to procurement, funding, trainings.
      3. Donors, like [PEPFAR or GLOBAL FUND]: changes to procurement, funding, trainings.
      4. Civil society organizations: changes to funding, outreach, locations where support is provided, advocacy.
   2. How has this varied by investment category? E.g. Saturation, sustained, central support
   3. Have there been any new laws or policies as a result of the geographic prioritization?
   4. Have there been any new ways to leverage funding as a result of the geographic prioritization?

1. What challenges have you faced in the last six months as a result of the geographic prioritization?
   1. Were these expected or unexpected challenges?
   2. How has your organization responded to these challenges?
   3. Have others faced similar challenges?
      1. How have they responded to these challenges?
   4. How likely is it that these challenges will be resolved?
2. How has your organization’s relationship with other stakeholders changed since May 2017 as a result of geographic prioritization?

*PROBE*: USG, MOH, implementing partners, other development partners/donors, local government, patient groups / civil society

1. What are the plans for the next 6-12 months of the geographic prioritization process?
   1. Who is implementing these plans?
   2. Who is playing a leadership role in carrying out these activities?
   3. What role will your organization play in those activities?
2. What plans has your organization discussed or put in place in case there are major shifts in disease burden in the future? E.g. rising prevalence in central support counties, moving counties into attained status.
   1. Do these plans include monitoring counties and facilities that have lost support? If so, please describe.
   2. What prompted the development of these plans?
   3. Who was involved?
   4. Have these plans been formalized in any documents or similar? E.g. Global Fund application, policy or strategy document, guidelines, etc.
   5. How often are these plans revisited?
   6. *IF NO PLANS DISCUSSED OR IN PLACE:* Has your organization discussed the possibility of putting a contingency plan in place? Why or why not?
3. In your view what else should have been done prior to the prioritization in order to help with the process, which was not done?
   1. How would this have helped?

1. Is there anything else significant about how the prioritization process is taking place that we should know about?

***FOR 4Cs PROJECT FOR OVC TRANSITION 🡪 ALL OTHERS SKIP TO END:***

1. What challenges did your project face in the last six months of implementing the transition preparation process for OVC programming?
   1. Were these expected or unexpected challenges?
   2. How did your organization responded to these challenges?
   3. Were these challenges resolved / Could they be resolved in the future?
2. What effect has the Geographic Prioritization / transition had on programming for Orphans and Vulnerable Children in the counties you supported?
   1. How well have OVC services been absorbed by local actors?
   2. How have different organizations changed the way they operated around OVC programming as a result of Prioritization / transition?

*PROBE*: County governments, national government agencies, donors/external partners, civil society, others.

- 1. What else could have been done to prepare for Geographic Prioritization / transition of OVC programming?

**Thank you for your time and contribution**
