# Supplementary material for: Effects of transition on HIV and non-HIV services and health systems in Kenya: a mixed methods evaluation of donor transition
Source: BMC Health Serv Res. 2021 May 13;21:457. doi: 10.1186/s12913-021-06451-y (PMC8117613; doi:10.1186/s12913-021-06451-y)
Supplement: Supplementary file 2 — Additional file 2. [file 12913_2021_6451_MOESM2_ESM.zip › SOAR_Comp1_IDI Guide_2016 07 08 v2R2.docx]

Project SOAR – Documenting the PEPFAR Geographic Prioritization

Semi-Structured Interview Guide – National-level Stakeholders

# Introduction

Thank you for agreeing to meet us.

We are conducting an assessment of PEPFAR’s geographic prioritization process; that is, the process through which counties/districts and sites fall into different investment categories. We are interested in the processes that took place before prioritization to prepare and after prioritization to implement the changes. Our goal is to provide practical information to local and national government, PEPFAR and other partners about how the geographic prioritization process took place and whether it has affected how services are delivered.

As part of the overall evaluation, we are documenting how the prioritization process has unfolded over time. We are interviewing various stakeholders about this process over the next 18-24 months to understand what decisions are being made about the prioritization, how these are communicated, what changes are made to plans as they are implemented, etc.

| Name of Organization |  |
| --- | --- |
| Your name |  |
| Designation |  |
| Work Area |  |
| Postal address |  |
| Telephone |  |
| E-mail address |  |

**OBTAIN INFORMED CONSENT**

*NOTE TO INTERVIEWER: This is a guide to the interview. You should cover* ***all the main numbered questions*** *in this interview form. You should use the probes selectively, according to the type of knowledge that the respondent conveys, and what you have already found out from documents and other interviews.*

# Interview Questions

## Geographic Prioritization Decisions / Awareness / Internal procedures

*INTERVIEWER*: First, I’m going to start by asking questions about how decisions about PEPFAR’s geographic prioritization were made.

1. Can you tell me a little about your current role?
2. How familiar are you with PEPFAR’s geographic prioritization process currently underway?
   1. When did you become aware that prioritization between counties/districts was taking place?
   2. How did you become aware of geographic prioritization? Who told you?
   3. What aspects of the geographic prioritization process, if any, have you been involved with thus far and to what extent?

*INTERVIEWER*: If respondent is not familiar with PEPFAR’s geographic prioritization, ask the respondent whether there is someone who is familiar with the process who can be interviewed instead.

1. Can you explain to me how decisions were made about how to allocate counties/districts or sites between investment categories?

*PROBE*: Evidence/data used; stakeholders involved, etc.

- 1. When were these decisions made?
  2. Have the allocations between categories changed over time? If so, how and why?
  3. Who explained the process to you and your colleagues?

1. How were other stakeholders informed about PEPFAR’s geographic prioritization?

*PROBE*: private / public meetings, directly from USG or MOH, etc.

- 1. How did this vary by stakeholders?

*PROBE*: national government (MOH, national AIDS control orgs), other donors/development partners, implementing partners, local government (county/district), civil society / patient group

1. How was the implementation of the PEPFAR’s geographic prioritization planned? / NON-USG: Were you aware of implementation plans?
   1. How did this plan change over time?
   2. How have key stakeholders communicated about the geographic prioritization?
   3. How were various stakeholders engaged in discussions/planning the process of implementing PEPFAR’s geographic prioritization? How are they currently engaged?
   4. What current structures are there in place to engage between PEPFAR and other stakeholders around transition? E.g. Transition team.
   5. What role, if any do you/your organization play?

## Geographic Prioritization Implementation

*INTERVIEWER:* Now I’m going to ask you about how PEPFAR’s geographic prioritization has been implemented thus far.

1. What did your organization do to prepare itself for the geographic prioritization?
   1. Have there been any management changes specifically related to prioritization?
   2. Have there been any funding changes specifically related to the prioritization?
2. What support has been provided to counties/districts or sites in order to prepare for the prioritization process?
   1. Were there specific activities that were done to prepare?
   2. Who provided most of the support to get the facility ready for transition?
   3. How did these entities support facilities to prepare for transition?
3. How is the geographic prioritization being implemented?
   1. What specific activities have taken place to roll out the prioritization?
   2. Are there management structures in place to support the prioritization? E.g. Transition team
      1. PROBE: management at various levels of health systems or in MOH vs. USG
   3. Are there specific funds available to support the roll out of prioritization? Who provided the funds?
      1. PROBE: PEPFAR, Government, other donors.
   4. How does this vary by investment category? E.g. Saturation, sustained, central support
4. How has the support that counties/districts and sites received for HIV services changed since prioritization began? E.g. staff hiring and salaries, commodities, training, funding, support for reporting, patient incentives, etc.
   1. How has this varied by service type? E.g. testing, treatment, prevention
   2. Any changes to how support is provided?
   3. Were all supported areas shifted at the same time?
   4. How has this varied by investment category? E.g. Saturation, sustained, central support
5. How has the support that counties/districts and sites received for non-HIV services, such as maternal and child health, changed since prioritization began? E.g. staff hiring and salaries, commodities, training, funding, support for reporting, patient incentives, etc.
   1. How has this varied by service type? E.g. antenatal care, family planning, immunization, malaria
   2. Any changes to how support is provided?
   3. Were all supported areas shifted at the same time?
   4. How has this varied by investment category? E.g. Saturation, sustained, central support
6. For those areas that are no longer supported by PEPFAR, how are these supported now?

[*IF PEPFAR SUPPORT HAS NOT ENDED AT TIME OF INTERVIEW:* What are the plans for support after PEPFAR ends its support?]

- 1. Which organization is supporting them?
  2. How was this decided?
  3. How well is it working?
  4. How do you expect this support to look like in the near future? In the mid-term?

## Effects of Geographic Prioritization

*INTERVIEWER:* Now I would like to ask you a series of questions about the effects of PEPFAR’s geographic prioritization thus far.

1. What effect has the prioritization had on HIV service delivery?
   1. How has the prioritization affected service coverage?
   2. How has the prioritization affected:
      1. Staff turnover?
      2. Staff motivation or performance?
      3. Availability of commodities?
      4. Reporting of data?
   3. How has the prioritization affected service quality and patient satisfaction?
2. What effect has the prioritization had on non-HIV service delivery, such as maternal and child health services?
   1. How has the prioritization affected service coverage?
   2. How has the prioritization affected:
      1. Staff turnover?
      2. Staff motivation or performance?
      3. Availability of commodities?
      4. Reporting of data?
   3. How has the prioritization affected service quality and patient satisfaction?
3. How have different organizations changed the way they operated as a result of PEPFAR’s geographic prioritization?
   1. Examples:
      1. Counties/districts or facilities: changes to reporting, staffing, etc.
      2. National government, like [NATIONAL AIDS CONTROL ORGANIZATION]: changes to procurement, funding, trainings.
      3. Donors, like [PEPFAR or GLOBAL FUND]: changes to procurement, funding, trainings.
   2. How has this varied by investment category? E.g. Saturation, sustained, central support
4. How has the broader health system responded to the geographic prioritization? E.g. new laws or policies, new ways to leverage funding, changes to the role of civil society, etc.
   1. How has this varied between national and local levels?
5. What challenges have you faced while implementing the geographic prioritization?
   1. Were these expected or unexpected challenges?
   2. How has your organization responded to these challenges?
   3. Have others faced similar challenges?
      1. How have they responded to these challenges?
   4. How likely is it that these challenges will be resolved?
6. How has your organization’s relationship with other stakeholders changed since prioritization started?

*PROBE*: USG, MOH, implementing partners, other development partners/donors, local government, patient groups / civil society

- 1. Have you collaborated or coordinated efforts with other stakeholders on the implementation of the geographic prioritization?
     1. If so, what have you collaborated or coordinated around?

1. In your view what else should have been done prior to the prioritization in order to help with the process, which was not done?
   1. How would this have helped?

1. What are the plans for the next 6-12 months of the geographic prioritization process?
   1. Who is implementing these plans?
   2. Who is playing a leadership role in carrying out these activities?
   3. What role will your organization play in those activities?
2. Is there anything else significant about how the prioritization process is taking place that we should know about?

**Thank you for your time and contribution**
