# Supplementary material for: Effects of transition on HIV and non-HIV services and health systems in Kenya: a mixed methods evaluation of donor transition
Source: BMC Health Serv Res. 2021 May 13;21:457. doi: 10.1186/s12913-021-06451-y (PMC8117613; doi:10.1186/s12913-021-06451-y)
Supplement: Supplementary file 3 — Additional file 3. [file 12913_2021_6451_MOESM3_ESM.docx]

# PEPFAR Geographic Prioritization Facility Survey

# Introduction

Thank you for agreeing to meet us.

We are conducting an assessment of PEPFAR’s geographic prioritization process; that is, the process through which sites have transitioned from receiving support from a PEPFAR implementing partner (IP) to central support. We are interested in learning about the kind of support that you have received from the PEPFAR IP, and how this is changing as PEPFAR transitions away from this facility. Our goal is to provide practical information to local and national government, PEPFAR and other partners about how the transition process took place and whether it has affected how services –including both HIV and non-HIV services - are delivered.

As part of the overall evaluation, we are conducting facility surveys. We are conducting one cross sectional survey across transition and sustain counties in Kenya/Uganda. The survey will cover service delivery, infrastructure and logistics support, supervision, drugs and commodities support, human resources, finance and budget, and transition arrangements.

# Section 0: Facility Information

| *To be prefilled by enumerator but checked at site:*   - 1. Facility County   2. Facility Name:   3. Facility GPS coordinates (if available)   4. Facility Level   5. Facility phone number:   6. Facility Ownership: |
| --- |

- 1. **In-charge Characteristics:**

| 0.7.1 Name: |  |
| --- | --- |
| 0.7.2 Cadre: |  |
| 0.7.3 Position |  |
| 0.7.4 Highest level of EDUCATION: |  |
| 0.7.5 Date STARTED AT CURRENT FACILITY: |  |
| 0.7.6 Gender: |  |
| 0.7.7 Contact Information: |  |

- 1. **Other Staff/Back-up Contact:**

| 0.8.1 Name: |  |
| --- | --- |
| 0.8.2 Cadre: |  |
| 0.8.3 PosiTION |  |
| 0.8.4 highest level of EDUCATION: |  |
| 0.8.5 dated started at CURRENT Facility: |  |
| 0.8.6 GENDER: |  |
| 0.8.7 Contact Information: |  |

# Section 1: Transition Arrangements

*Instructions to Enumerator:*

*For HC II or III, address questions for this section to the facility in-charge.*

*For HC IV or Hospital, ask to speak to the head of the HIV unit for this section.*

- 1. *Transition preparedness*
     1. Which APHIA Program [is/was] providing support to your facility?
     2. Has your facility stopped receiving support from the APHIA Program for your HIV services or HIV program?
     3. Did you receive an official date by which the APHIA Program would withdraw support? If so, what was that date? Select the month and year

Enumerator instructions: if facility has not transitioned, use the default date of September 1, 2016

- - 1. When did you actually stop receiving support from the APHIA Program?

***Please ask the following questions ONLY to facilities that have lost APHIA Program/PEPFAR support***

***For facilities that have not yet lost APHIA Program/PEPFAR support, please skip to 1.1.10***

- - 1. Did you receive any communication that your facility would no longer be receiving APHIA Program support?
       1. If YES, when did you first learn that this facility would no longer receive support from the APHIA Program?
    2. Who first informed the facility that it would be transitioning from APHIA Program support?
  1. MoH (National)
  2. NASCOP/NACC
  3. County Government
  4. APHIA Program or PEPFAR IP
  5. USAID or other USG agency
  6. NGO or company that owns facility
  7. Association to which facility belongs (eg. Church Medical Association)
  8. Other (Specify)
  9. No one/we did not receive explicit notice that the facility would be transitioning from APHAI Program/PEPFAR support
     1. Did you have a strategy for how to cope with the withdrawal of APHAI Program support? Yes/no

***For facilities that do NOT have a strategy skip to Q1.1.9***

- - 1. If yes, which of the following actors have provided your facility with support for developing this strategy (Select all that apply):

| **Actors** | **Yes/No** |
| --- | --- |
| MoH (National) |  |
| NASCOP/NACC |  |
| County Government |  |
| APHIA Program |  |
| USAID or other USG agency (e.g. Capacity) |  |
| NGO or company that owns facility |  |
| Association to which facility belongs (eg. Church Medical Association) |  |
| Other Donor Agency [specify] |  |
| Other [specify] |  |

- - 1. Please indicate the degree to which you agree or disagree with the following statements, using a scale where “6” represents strongly agree and “1” represents strongly disagree.

| **Preparedness components** | Strongly agree (5) | Agree (4) | Neither agree nor disagree (3) | Disagree (2) | Strongly disagree (1) | Don’t know/not applicable |
| --- | --- | --- | --- | --- | --- | --- |
| **Communication** |  |  |  |  |  |  |
| 1.1.9.1 All staff were properly informed about the transition from Aphia Program support |  |  |  |  |  |  |
| 1.1.9.2 Our facility was able to communicate adequately with our patients and communities about shifts in service related to the transition |  |  |  |  |  |  |
| **Alignment** |  |  |  |  |  |  |
| 1.1.9.3 All health workers that previously were hired or paid by the Aphia Program were retained at this facility in their same post |  |  |  |  |  |  |
| 1.1.9.4 Our facility did not have to change the way HIV services were delivered as a result of transition |  |  |  |  |  |  |
| 1.1.9.5 Our facility did not have to change the way services for KPs are delivered as a result of transition |  |  |  |  |  |  |
| 1.1.9.6 Our facility did not have to change the way MNCH services are delivered as a result of transition |  |  |  |  |  |  |
| 1.1.9.7 Our facility had to coordinate with neighboring facilities about filling gaps in services as a result of transition |  |  |  |  |  |  |
| 1.1.9.8 Our facility did not have to change reporting mechanisms as a result of transition |  |  |  |  |  |  |
| **Capacity** |  |  |  |  |  |  |
| 1.1.9.9 Our facility was adequately prepared to transition from Aphia Program support |  |  |  |  |  |  |
| 1.1.9.10 Our facility management and leadership were equipped with sufficient knowledge and skills to prepare for the transition |  |  |  |  |  |  |
| 1.1.9.11 Our facility staff were equipped with sufficient knowledge and skills to prepare for the transition |  |  |  |  |  |  |

***Please ask the following questions ONLY to facilities that have not yet lost PEPFAR support***

- - 1. Has there ever been any suggestion, from your IP, or from government that you might in the future lose support from the Aphia Program? Yes/No

If “No” then skip to 1.1.12

- - 1. If yes, do you have a strategy to help you prepare for the potential withdrawal of Aphia Program/PEPFAR IP support? Yes/No

***For both facilities that have transitioned, and those that have not yet transitioned but have been told that they may do so:***

- - 1. Have you ever received any support (i.e. training, technical assistance, cash support, other) to prepare the facility for (potential) transition?

YES/NO

If “NO” skip to 1.2

|  | 1.1.12.1 What type of support did you receive? | 1.1.12.2 From whom did you receive this support? |
| --- | --- | --- |
|  | Tick all that apply (prompt respondent to list all types of support provided) | MoH (National), County Government, APHIA Program or PEPFAR IP, USAID or other USG agency, NGO or company that owns facility, Association to which facility belongs, Other Donor Agency (specify), Other (specify) |
| Cash support |  |  |
| Training |  |  |
| Technical assistance |  |  |

***For ALL FACILITIES***

- 1. *Changes in key services*

I am going to read out a series of statements, I would like you to complete these sentences for me using a scale of 1-5, where:-

| 1 | Got much worse |
| --- | --- |
| 2 | Got worse |
| 3 | Stayed the same |
| 4 | Got better |
| 5 | Got much better |

|  |  | Score (1-5) | If score is (1) or (5) then why is this the case? |
| --- | --- | --- | --- |
| 1.2.1 | Since (ENTER TRANSITION DATE/September 1, 2016) the ability of the average patient to access HIV/AIDS services in our catchment area has got ……… |  |  |
| 1.2.2 | Since (ENTER TRANSITION DATE/September 1, 2016) the ability of the poorest and most marginalized patients in our catchment area to access HIV/AIDS services has got ……… |  |  |
| 1.2.3 | Since (ENTER TRANSITION DATE/September 1, 2016) ] the ability of key populations to access HIV/AIDS care and treatment services in our catchment area has got ……… |  |  |
| 1.2.4 | Since (ENTER TRANSITION DATE/September 1, 2016) the quality of HIV/AIDS services in this facility has got …… |  |  |
| 1.2.5 | Since (ENTER TRANSITION DATE/September 1, 2016) the ability of the average patient to access MNCH services in our catchment area has got ……… |  |  |
| 1.2.6 | Since (ENTER TRANSITION DATE/September 1, 2016) the ability of the poorest and most marginalized patients in our catchment area to access MNCH services in this area has got ……… |  |  |
| 1.2.7 | Since (ENTER TRANSITION DATE/September 1, 2016) ] the quality of MNCH services in this facility has got …… |  |  |

1.3 What are your TOP 3 biggest concerns about the effects of the (possible) withdrawal of APHIA Program/PEPFAR IP support to this facility? *Instructions to Enumerator: Allow respondent to speak freely, and then identify the options that correspond most closely to the respondent’s answer.*

a. Financial concerns

b. Drug and commodity supplies

c. Human Resources

d. Patient Access

e. Lack of patient follow-up

f. Lack of support for support groups

g. Lack of supervision

h. Lack of technical assistance (e.g. training on new guidelines)

i. Lack of tools (e.g. registers, forms, stationary)

j. Other (specify)

1.4 Impact of transition on facility operations ***(for facilities that have already transitioned ONLY)***

I am going to read out a series of statements about how the withdrawal of PEPFAR IP support to this facility might affect facility operations, for each statement I would like you to tell me whether:-

- The effect has already occurred
- The effect has not yet occurred but you expect it to do so
- You don’t expect this effect to occur
- You don’t know whether the effect is likely to occur or not

| Expected effects | a.Answer options:  ~Already occurred  ~ Expect to occur  ~ Don’t expect  ~ Don’t know |
| --- | --- |
| 1.4.1.Staff spend less time in trainings required by the APHIA program and will have more time to focus on patient services or other health facility activities |  |
| 1.4.2 Staff have more time to spend on MNCH services |  |
| 1.4.3 County Health Office provide the support needed for support supervision, mentoring staff etc. |  |
| 1.4.4 County health officers or facility management are more engaged in day-to-day facility operations? |  |
| 1.4.5 Staff spend less time on HIV services |  |
| 1.4.6 Delays occur in DHIS2/iHRIS data submission |  |
| 1.4.7 Delays occur in drug and supply orders |  |
| 1.4.8 There are stock outs in lab/unable to run tests |  |
| 1.4.9 Lack of access to specialized testing due to loss of support for transport |  |
| 1.4.10 Inability to attend trainings/cascade learnings from trainings |  |
| 1.4.11 Loss of volunteers and contract staff |  |
| 1.4.12 Loss of staff through their resignation, or reassignment to other health facilities |  |
| 1.4.13 Increased absenteeism among staff due to loss of incentives |  |
| 1.4.14 Reduced morale and productivity of staff due to loss of incentives |  |
| 1.4.15 Patients must choose to go elsewhere for care |  |

# Section 2: Service Delivery

- 1. Select the services which are offered at your facility and indicate the support you have received for the following:

| **List of services** | 2.1.1 Do you currently offer this service at your facility? (Y/N) | 2.1.2 If YES, who is the **primary** organization offering support to this service? (MoH (National), County Government, APHIA Program or PEPFAR IP, USAID or other USG Agency, NGO or company that owns facility, Association to which facility belongs, Other, No One) | 2.1.3 Did you formerly offer this service before TRANSITION DATE? | 2.1.4 If YES, who was the **primary** organization providing support prior to TRANSITION DATE? MoH (National), County Government, APHIA Program or PEPFAR IP, USAID or other USG Agency, NGO or company that owns facility, Association to which facility belongs, Other, No one) | 2.1.5 If NO to 2.1.1 and YES to 2.1.3: Did this service end on TRANSITION DATE? | 2.1.6 If NO to 2.1.5, when did this service ACTUALLY end? | 2.1.7 If NO to 2.1.1 and YES to 2.1.3, Why did this service end? (High level policy shift, contract ended, etc) | 2.1.8  If YES to 2.1.1, and YES to 2.1.3: has service quality changed since TRANSITION DATE? (Improve, Decline, Stay the Same) |
| --- | --- | --- | --- | --- | --- | --- | --- | --- |
| **Part 1: HIV services** | | | | | | |  |  |
| ART |  |  |  |  |  |  |  |  |
| Outreach services |  |  |  |  |  |  |  |  |
| PMTCT |  |  |  |  |  |  |  |  |
| HCT/VCT |  |  |  |  |  |  |  |  |
| **Part 2: MNCH/FP services** | | | | | | |  |  |
| Ante-natal care |  |  |  |  |  |  |  |  |
| Delivery |  |  |  |  |  |  |  |  |
| Immunizations |  |  |  |  |  |  |  |  |
| Nutrition |  |  |  |  |  |  |  |  |
| Child Welfare Clinic |  |  |  |  |  |  |  |  |

- 1. Has your facility started to offer any new services since TRANSITION DATE? Yes/No

If yes

| **2.2.1 Name of service** | **2.2.2 Do you receive any support (financial or technical) from agencies outside of this facility, for this service? (yes/no)** | **2.2.3 If Yes, please specify who provides support**  (National), County Government, APHIA Program or PEPFAR IP (specify), USAID or other USG Agency, NGO or company that owns facility, Association to which facility belongs, Other (specify), No One) |
| --- | --- | --- |
|  |  |  |
|  |  |  |
|  |  |  |

# Section 3: Drugs, Commodities and Laboratory Support

*Components: Drugs, commodities and lab support (e.g. frequency of supply of drugs and other commodities for HIV and non-HIV, support to ordering for drugs and commodities, availability and stock outs, quality of drugs and commodities, access to lab services)*

3.1 During the past year has anyone or any organization, including the government, provided you with any support related to drugs and/or other commodities relevant to your **HIV/AIDS services?** Please note that KEMSA is considered a part of the MoH (National).

(YES/NO)

If No, skip to question 3.2.

| **HIV SERVICES** | **If Yes,**  3.1.1 Have you received support for your HIV/AIDS services in relation to drugs and commodities from [APHIA Program, MoH (National), MoH (County), Other (Specify)]  3.1.2. What kind of support have you received in relation to drugs and commodities for your HIV/AIDS services from the [APHIA Program, MoH (National), MoH (County), Other (Specify)] | | | | | 3.1.3 Has support increased or decreased or stayed the same since [INSERT TRANSITION DATE/September 1, 2016] Skip if no support received. | 3.1.4 If support has decreased, how has the facility responded to change. |
| --- | --- | --- | --- | --- | --- | --- | --- |
|  | Direct provision of drugs/commodities | Quantification/Ordering | Purchasing | Delivering/Redistribution | Commodity Security (ensuring adequate stock) | ~INCREASE; ~DECREASE; ~ STAY THE SAME; ~DON'T KNOW | ~NO RESPONSE; ~SUBSTITUTED WITH OTHER SOURCE (SPECIFY); ~GONE WITHOUT; ~ADAPTED OPERATIONS TO ACCOMMODATE  ~OTHER (SPECIFY) |
| APHIA Program |  |  |  |  |  |  |  |
| MoH (National) |  |  |  |  |  |  |  |
| MoH (County) |  |  |  |  |  |  |  |
| Other (specify) |  |  |  |  |  |  |  |

3.2 During the past year has anyone or any organization, including the government, provided you with any support related to drugs and/or other commodities relevant to your **MNCH services?** Please note that KEMSA is considered a part of the MoH (National).

(YES/NO)

If No, skip to question 3.3

| **MNCH SERVICES** | **If Yes,**  3.2.1 What kind of support have you received (e.g. direct payments or direct provision of drugs/commodities; support with ordering, purchasing, and/or delivery)?  3.2.2. Who provided you with this support?  *Please mark X in the appropriate cell in the table below.* | | | | 3.2.3 Has support increased or decreased or stayed the same since [INSERT TRANSITION DATE September 1, 2016] Skip if no support received | 3.2.4 How has the facility responded to change |
| --- | --- | --- | --- | --- | --- | --- |
|  | Direct payments or provision | Ordering | Purchasing | Delivering | ~INCREASE; ~DECREASE; ~ STAY THE SAME; ~DON'T KNOW | ~NO RESPONSE; ~SUBSTITUTED WITH OTHER SOURCE (SPECIFY); ~GONE WITHOUT; ~ADAPTED OPERATIONS TO ACCOMMODATE  ~OTHER (SPECIFY) |
| APHIA Program) |  |  |  |  |  |  |
| MoH (National) |  |  |  |  |  |  |
| MoH (County) |  |  |  |  |  |  |
| Other (Specify) |  |  |  |  |  |  |

- 1. Please provide details about the availability, quality, and price of the following tracer drugs and/or commodities.

| Name of drug or commodities | - - 1. Do you currently dispense these at your facility? (YES/NO)     If NO, go to 3.3.2  If YES, go to 3.3.2 | - - 1. Did you formerly dispense these before Transition Date/September 1, 2016?   If “NO” to 3.3.1 AND 3.3.2 🡪 next drug  3.3.1 YES 🡪 3.3.2 NO/YES 🡪 3.3.4  3.3.1 NO 🡪 3.3.2 YES 🡪 3.3.3 🡪 Next drug | 3.3.3 Why did you stop dispensing this drugs since TRANSITION DATE/ September 1, 2016  (Possible choices:   - DP no longer supplying these - DP has begun support - High-level MOH policy change - Not available for purchase - No longer profitable - Out of stock - Lack of demand - Inadequate staff - Don’t know - Other | 3.3.4 IF YES to 3.3.1 Is this drug currently in stock today? (YES/NO) | 3.3.5 If NO to 3.3.4, mark how many days out of stock (How many days ago was it last in stock) | 3.3.6 How has the frequency of stock-outs of this drug changed since the transition period?  Choices:  - increased  - decreased  - no change  - don’t know |
| --- | --- | --- | --- | --- | --- | --- |
| **Part 1: HIV** |  |  |  |  |  |  |
| ARV First line   - TDF/3TC/(EFV or NVP) - AZT/3TC/NVP |  |  |  |  |  |  |
| 1^st^ line Anti-TB medicine   - HRZE - RH |  |  |  |  |  |  |
| Condoms (male) |  |  |  |  |  |  |
| Condoms (female) |  |  |  |  |  |  |
| **Part 2: MNCH** |  |  |  |  |  |  |
| First line drug for malaria (ACTs): AL |  |  |  |  |  |  |
| Cotrim® (*Cotrimoxazole*) |  |  |  |  |  |  |
| Amoxcyillin (respiratory tract infections) |  |  |  |  |  |  |
| DPT/Pentavalent (Immunization) |  |  |  |  |  |  |
| Iron Folic Acid |  |  |  |  |  |  |
| Depo-Provera or other injectable |  |  |  |  |  |  |

*Lab services support and availability*

- 1. Does your facility have a laboratory or offer laboratory services?

YES/NO

If Yes, Which of the following laboratory services do you typically offer at this facility?

| Name of lab test or service | 3.4.1 Do you currently conduct these tests and services at your facility? (YES/NO)  If NO go to 3.4.2  If YES go to 3.4.2 | 3.4.2 Did you formerly provide this lab service before TRANSITION DATE/September 1 2016?  If “NO” to 3.4.1 AND 3.4.2 🡪 next test/service  3.4.1 YES 🡪 3.4.2 NO/YES 🡪 3.4.4  3.4.1 NO 🡪 3.4.2 YES 🡪 3.4.3 🡪 Next Service | 3.4.3 Why did you stop conducting this service since TRANSITION DATE/September 1 2016?   - Shift to regional lab service - IP no longer Supporting this - High-level GOU policy change - Don’t know - Other | 3.4.4 If YES to 3.4.1, Are you able to conduct this test today?  YES/NO | 3.4.5 If NO to 3.4.4, how many days ago were you last able to conduct this test? | 3.4.6 How has the frequency of lab disruptions changed since the transition period?  Choices:  - increased  - decreased  - no change  - don’t know |
| --- | --- | --- | --- | --- | --- | --- |
| Rapid HIV antibody tests |  |  |  |  |  |  |
| Viral Load Counts |  |  |  |  |  |  |
| Sputum for smear microscopy (TB diagnosis) |  |  |  |  |  |  |
| RDTs for malaria |  |  |  |  |  |  |
| Peripheral blood smear (malaria diagnosis) |  |  |  |  |  |  |
| Rapid syphilis test |  |  |  |  |  |  |
| Urine microscopy |  |  |  |  |  |  |
| ANC Profile |  |  |  |  |  |  |
| Chest X-ray |  |  |  |  |  |  |

- 1. During the past year, has anyone or any organization, including the government, provided you with any laboratory support relevant to your HIV/AIDS services? Please note that KEMSA is considered a part of the MoH (National).

If “NO” skip to Section 3.6

| **HIV SERVICES** | **Lab support**  **If Yes,**  3.5.1 Have you received support for your HIV/AIDS services in relation to your laboratories from: [APHIA Program/MoH (National)/MoH (County)/Other (specify)]  3.5.2. What kind of support have you received in relation to your laboratories for your HIV/AIDS services? | | | | | | | | 3.5.3 Has support increased or decreased or stayed the same since [INSERT TRANSITION DATE/September 1, 2016] Skip if no support received | 3.5.4 How has the facility responded to change |
| --- | --- | --- | --- | --- | --- | --- | --- | --- | --- | --- |
|  | **Testing kits** | **Lab equipment & reagents** | **Transport of lab specimens to regional labs** | **Financial support for facilities to transport lab specimens to regional labs** | **Training** | **Counselors** | **Bringing or transporting results** | **Other (specify)** | **~INCREASE; ~DECREASE; ~ STAY THE SAME; ~DON'T KNOW** | **~NO RESPONSE; ~SUBSTITUTED WITH OTHER SOURCE (SPECIFY); ~GONE WITHOUT; ~ADAPTED OPERATIONS TO ACCOMMODATE**  **~OTHER (SPECIFY)** |
| APHIA Program or |  |  |  |  |  |  |  |  |  |  |
| MoH (National) |  |  |  |  |  |  |  |  |  |  |
| MoH (County) |  |  |  |  |  |  |  |  |  |  |
| Other (Specify) |  |  |  |  |  |  |  |  |  |  |

- 1. During the past year, has anyone or any organization, including the government, provided you with lab support relevant to your **MNCH services?** Please note that KEMSA is considered a part of the MoH (National)

(YES/NO)

If “NO” please skip to Section 4

| **MNCH SERVICES** | **Lab support**  **If Yes,**  3.6.1 What kind of support have you received) (e.g. testing kits, provision of lab equipment or reagents, transport for specimens to lab, or financial support to transport specimens)?  3.6.2. Who provided you with this support? | | | | | | | | 3.6.3 Has support increased or decreased or stayed the same since [INSERT TRANSITION DATE/September 1, 2016]. Skip if no support received | 3.6.4 How has the facility responded to change |
| --- | --- | --- | --- | --- | --- | --- | --- | --- | --- | --- |
|  | **Testing kits** | **Lab equipment & reagents** | **Transport of lab specimens to regional labs** | **Financial support for facilities to transport lab specimens to regional labs** | **Training** | **Counselors** | **Bringing or transporting results** | **Other (specify)** | **~INCREASE; ~DECREASE; ~ STAY THE SAME; ~DON'T KNOW** | **~NO RESPONSE; ~SUBSTITUTED WITH OTHER SOURCE (SPECIFY); ~GONE WITHOUT; ~ADAPTED OPERATIONS TO ACCOMMODATE**  **~OTHER (SPECIFY)** |
| APHIA Program |  |  |  |  |  |  |  |  |  |  |
| MoH (National) |  |  |  |  |  |  |  |  |  |  |
| MoH (County) |  |  |  |  |  |  |  |  |  |  |
| Other (Specify) |  |  |  |  |  |  |  |  |  |  |

# Section 4: Supervision

- 1. Do you receive supervision visits for HIV/MNCH services? YES/NO

|  | 4.1.1 What is the frequency with which your facility is supposed to receive supportive supervision visits? | 4.1.2 Prior to [ENTER TRANSITION DATE]/September 1, 2016, what was the actual frequency with which your facility actually received supportive supervision visits? | 4.1.3 Since [ENTER TRANSITION DATE/September 1, 2016] what is the actual frequency with which your facility actually receives supportive supervision visits? | 4.1.4 Who is a part of these supervisory visits? | 4.1.5 How helpful are these supervisory visits? |
| --- | --- | --- | --- | --- | --- |
|  | Monthly, quarterly, biannually | Monthly, quarterly, biannually | Monthly, quarterly, biannually | -District health team  -PEPFAR IP or Aphia Program  -Other | - Extremely helpful  - Very helpful  - Neither helpful nor unhelpful  -Somewhat helpful  - Not helpful at all |
| HIV services |  |  |  |  |  |
| MNCH services |  |  |  |  |  |

# Section 5: Human Resources

*Instructions to Enumerators: for HCIVs and Hospitals this question should be applied only to those in the HIV/AIDS clinic and/or MNCH ward.*

- 1. **Current Staffing:**

**Revised Sections on HR Support:**

Instructions to Enumerators:

1. If at a small facility (HC III/level 3 or lower), please address questions to facility in-charge. If at a higher-level facility, address questions to the director of the HIV clinic (or other outpatient clinics that provide clinical HIV care).
2. Request the names of all staff members who provide HIV services. For small facilities, ask the respondent to enumerate all clinical staff. For higher-level facilities, ask each director to enumerate the clinical staff that work in the HIV clinics, or who work in other clinics but provide HIV care.
3. Identify names for all filled positions, if a staff member is named more than once as supporting different services, please collect information from all sources on that person.
4. At the end of the interview(s), randomly select from the list of staff members who are present one person who primarily supports HIV services and one person who primarily supports MNCH. If there are no such persons, randomly select two names from the list of staff present. Tell the respondent(s), I would like to speak with these individuals privately to administer a 10-minute survey to them.

| **5.1.1 Please name all of the staff who provide HIV services** | **5.1.2 What is their cadre?** | **5.1.3 Who is currently paying the salary for this person? Select ALL sources of support** | **5.1.4 Who paid the salary prior to [INSERT TRANSITION DATE]?** | **5.1.5 Has there been turnover in this position since TRANSITION DATE?** | **5.1.6 Is the staff member present?** | **5.1.7 If not present, reason for not being present** |
| --- | --- | --- | --- | --- | --- | --- |
|  |  | **MOH (National), County Government, Facility, APHIA Program, APHIA Program through county, USAID or other USG Agency, NGO or company that owns facility, Association to which facility belongs, Facility, Individual does not receive a salary, Other (specify), Don’t Know** | **MOH (National), County Government, Facility, APHIA Program, APHIA Program through county, USAID or other USG Agency, NGO or company that owns facility, Association to which facility belongs, Facility, Individual does not receive a salary, Other (specify), Don’t Know** | **Yes/No** | **Yes/No** | **Outreach, travel to district, training, meeting with IP, sick leave, bereavement/funeral, scheduled leave, other** |
|  |  |  |  |  |  |  |
|  |  |  |  |  |  |  |
|  |  |  |  |  |  |  |
|  |  |  |  |  |  |  |
|  |  |  |  |  |  |  |
|  |  |  |  |  |  |  |
|  |  |  |  |  |  |  |
|  |  |  |  |  |  |  |
|  |  |  |  |  |  |  |
|  |  |  |  |  |  |  |
|  |  |  |  |  |  |  |
|  |  |  |  |  |  |  |
|  |  |  |  |  |  |  |
|  |  |  |  |  |  |  |

5.2 Do you have any vacant posts in your HIV Team?

- - 1. IF YES, how many?
  1. Since TRANSITION DATE/September 2016, have you had to terminate any positions as a result of losing support from the APHIA Program?
     1. How many posts have you terminated?
     2. What cadres of staff were terminated?

**5.4 Training**

5.4.1 Have you or any of your staff members who provide HIV services attended a training since TRANSITION DATE?

Yes/no

If “NO” skip to Section 6.

| **5.4.2 What topics did the training cover?** | **5.4.3 How many staff attended the training?** | **5.4.4 What cadres of staff attended the training?** | **5.4.5 How long was the training?** | **5.4.6 Who provided the training? Select ALL that apply** |
| --- | --- | --- | --- | --- |
| **ART, HIV Clinical Care, PMTCT, VMMC, TB, IMCI, MCH, STIs, Family Planning, Malaria, Reporting, Other (specify)** |  |  | **(days)** | **MOH (National), County Government, APHIA Program or PEPFAR IP, USAID or other USG Agency, NGO or company that owns facility, Association to which facility belongs, Other (specify), No One** |
|  |  |  |  |  |
|  |  |  |  |  |

# Section 6: Finance and Budget

**Direct Budget and Revenue figures:**

*Instructions to Enumerator: Ask to see the facility financial records and complete these tables based on the records in consultation with the respondent.*

6.1 Facility Budget

What is the facility budget for the current and previous fiscal/budget years?

|  | Dates | Amount in Kenyan Shillings |
| --- | --- | --- |
| Current fiscal year |  |  |
| Previous fiscal year |  |  |

6.2 Funds released and source

|  | | | **Total:** | **6.2.2 Of this amount, how much was received from each of the following sources?** | | | | | | | | |
| --- | --- | --- | --- | --- | --- | --- | --- | --- | --- | --- | --- | --- |
| **Year** | **Fiscal Quarter** | **Month** | **6.2.1 What amount was actually released to the facility in the prior 15 months (or five quarters)** | **County Government** | **National MoH** | **National Health Insurance Fund (NHIF)** | **Patient Fees** | **Private (Investmnet, Donation)** | **APHIA Program** | **Other Donor (e.g. UNICEF, Global Fund, JICA, Danida, WB, other countr gov, uN agencies)** | **NGO, Community, Other** | **Total** |
| 2016 | Q3 | Jan |  |  |  |  |  |  |  |  |  |  |
|  |  | Feb |  |  |  |  |  |  |  |  |  |  |
|  |  | Mar |  |  |  |  |  |  |  |  |  |  |
|  | Q4 | April |  |  |  |  |  |  |  |  |  |  |
|  |  | May |  |  |  |  |  |  |  |  |  |  |
|  |  | June |  |  |  |  |  |  |  |  |  |  |
|  | Q1 | July |  |  |  |  |  |  |  |  |  |  |
|  |  | Aug |  |  |  |  |  |  |  |  |  |  |
|  |  | Sept |  |  |  |  |  |  |  |  |  |  |
|  | Q2 | Oct |  |  |  |  |  |  |  |  |  |  |
|  |  | Nov |  |  |  |  |  |  |  |  |  |  |
|  |  | Dec |  |  |  |  |  |  |  |  |  |  |
| 2017 | Q3 | Jan |  |  |  |  |  |  |  |  |  |  |
|  |  | Feb |  |  |  |  |  |  |  |  |  |  |
|  |  | Mar |  |  |  |  |  |  |  |  |  |  |
|  |  | TOTAL |  |  |  |  |  |  |  |  |  |  |

6.3 Facility expenditure - How much did the facility spend (by month/quarter) on each of the following items?

| **Year** | **Fiscal Quarter** | **Month** | **Salaries**  **/wages** | **Staff bonuses** | **Staff allowances** | **Utilities & rent** | **Maintenance & repairs** | **Drugs & commodities** | **Equipment** | **Office supplies and printing** | **Taxes & tariffs** | **Food for patients** | **Invest-ments** | **Other** | **Total** |
| --- | --- | --- | --- | --- | --- | --- | --- | --- | --- | --- | --- | --- | --- | --- | --- |
| 2016 | Q3 | Jan |  |  |  |  |  |  |  |  |  |  |  |  |  |
|  |  | Feb |  |  |  |  |  |  |  |  |  |  |  |  |  |
|  |  | Mar |  |  |  |  |  |  |  |  |  |  |  |  |  |
|  | Q4 | April |  |  |  |  |  |  |  |  |  |  |  |  |  |
|  |  | May |  |  |  |  |  |  |  |  |  |  |  |  |  |
|  |  | June |  |  |  |  |  |  |  |  |  |  |  |  |  |
|  | Q1 | July |  |  |  |  |  |  |  |  |  |  |  |  |  |
|  |  | Aug |  |  |  |  |  |  |  |  |  |  |  |  |  |
|  |  | Sept |  |  |  |  |  |  |  |  |  |  |  |  |  |
|  | Q2 | Oct |  |  |  |  |  |  |  |  |  |  |  |  |  |
|  |  | Nov |  |  |  |  |  |  |  |  |  |  |  |  |  |
|  |  | Dec |  |  |  |  |  |  |  |  |  |  |  |  |  |
| 2017 | Q3 | Jan |  |  |  |  |  |  |  |  |  |  |  |  |  |
|  |  | Feb |  |  |  |  |  |  |  |  |  |  |  |  |  |
|  |  | Mar |  |  |  |  |  |  |  |  |  |  |  |  |  |
|  |  | TOTAL |  |  |  |  |  |  |  |  |  |  |  |  |  |

6.4.1 Comparing the period prior to (ENTER TRANSITION DATE/September 1, 2016) with the current period, would you say that the facility is better off, worse off or in the same position financially?

Better/worse/same

6.4.2 Why is the facility better off/worse off/ the same? (open-ended)

_________________________________________

***Part A of the survey is now finished. Thank the respondent for their cooperation and move to Part B, the individual questionnaires.***

# Part B - Individual questionnaires

Note to enumerator: these individual questionnaires should be applied to the individuals identified in question 5.1 above. In HC II and IIIs these questions should also be applied to the in-charge

**INTRODUCTION**

7.1 Respondent details

| 7.1.1 Name: |
| --- |
| 7.1.2 Cadre: |
| 7.1.3 Position: |
| 7.1.4 Highest Level of Education: |
| 7.1.5 Date Started Current Position:  7.1.6 Gender: |

**7.2 Time allocation**

**For each question, indicate whether efforts have increased, decreased or stayed the same since TRANSITION DATE.**

7.2.1 Since TRANSITION DATE, how has the average amount of time you spend on HIV clinical care changed?

7.2.2 Since TRANSITION DATE, how has the average amount of time you spend on non-HIV clinical care changed?

7.2.3 Since TRANSITION DATE, how has the average amount of time you spend on preparing reports changed?

7.2.4 Since TRANSITION DATE, how has the average amount of time you spend on meetings changed?

7.2.5 Since TRANSITION DATE, how has the average amount of time you spend on trainings changed?

7.2.6 Since TRANSITION DATE, how has the average amount of time you spend on administrative duties (e.g. budget, payroll), changed?

7.2.7 Since TRANSITION DATE, how has the average amount of time you spend on other activities changed?

7.3 Other forms of support

| **7.3.1 Besides salary, did you receive additional support intended for HIV care prior to [INSERT TRANSITION DATE]?** | | | **7.3.2 Who provided this additional support for HIV care? (Skip if no support given)** | **7.3.3 Overall, has support for HIV care from this source increased/decreased or stayed the same since [INSERT TRANSITION DATE]?** |
| --- | --- | --- | --- | --- |
| **Bonuses, top-ups,** | **Outreach Allowances**  **Yes/No/DK** | **Other**  **(Specify)** | **(All that apply)** |  |
| **Yes/No/DK** | **Yes/No/DK** | **Yes/No/DK** | **MOH (National), MoH (County), APHIA Program, APHIA Program through county, USAID or other USG agency, NGO or company that owns facility, Association to which facility belongs, Facility, this individual does not receive a salary, Other (specify), Don’t Know** | **~INCREASED; ~DECREASED; ~ STAYED THE SAME; ~DON'T KNOW** |

**Motivation and satisfaction**

I am going to read out a series of statements, on a scale of 1 -5 where

| 1 | Strongly disagree |
| --- | --- |
| 2 | Disagree |
| 3 | Neither agree nor disagree |
| 4 | Agree |
| 5 | Strongly agree |

Can you tell me how strongly you agree or disagree with each of the following statements:

| 7.4 10-item Motivation Index (adapted from Mbindyo et al. 2009): | | 1 Strongly disagree  2 Disagree  3 Neither agree nor disagree  4 Agree  5 Strongly agree |
| --- | --- | --- |
| 7.4.1 | I feel motivated to work hard |  |
| 7.4.2 | Overall, I am very satisfied with my job |  |
| 7.4.3 | I am satisfied with the opportunity to use my abilities in my job |  |
| 7.4.4 | This job makes me feel good about myself |  |
| 7.4.5 | I am proud to be working for this facility |  |
| 7.4.6 | I am glad that I work for this facility rather than other facilities in the country |  |
| 7.4.7 | The facility really inspires me to do my very best on the job |  |
| 7.4.8 | I always complete by tasks efficiently and correctly |  |
| 7.4.9 | I am a hard worker |  |
| 7.4.10 | I am punctual about coming to work |  |

7.5 Over the past month how satisfied have you been in your job at this facility?

1 – extremely dissatisfied

1. – dissatisfied
2. – neither satisfied nor dissatisfied
3. – satisfied
4. – extremely dissatisfied

7.6 Since [Transition Date/September 1, 2016], how has your satisfaction with your job changed? _____

| 1 | Got much worse |
| --- | --- |
| 2 | Got worse |
| 3 | Stayed the same |
| 4 | Got better |
| 5 | Got much better |

THANK YOU VERY MUCH!
