# Supplementary material for: Effects of transition on HIV and non-HIV services and health systems in Kenya: a mixed methods evaluation of donor transition
Source: BMC Health Serv Res. 2021 May 13;21:457. doi: 10.1186/s12913-021-06451-y (PMC8117613; doi:10.1186/s12913-021-06451-y)
Supplement: Supplementary file 4 — Additional file 4. [file 12913_2021_6451_MOESM4_ESM.zip › SOAR_Comp3_IntGuides CS R1R2.pdf]

In-Depth Interview Guide #1 (Facility In-Charge)

Project SOAR – Longitudinal Case Studies of PEPFAR Geographic Prioritization

INTERVIEW QUESTIONS

1. Can you tell me a little about your current role, and how familiar you are with this facility?

INTERVIEWER: If the respondent does not seem at all familiar with the case study facility, then please ask if there is someone else who is more familiar with the facility whom you could talk to.

2. Are you familiar with the recent removal of APHIA Program support and transition of the facility to government support?

   PROBE: Have you had any recent changes in how [APHIA PROGRAM] has supported the facility?

   a. Were you involved at all in this transition process at this facility?

INTERVIEWER: If the respondent does not seem at all familiar with transition, then please ask if there is someone else who is more familiar with transition whom you could talk to.

3. Can you tell me about what types of support this facility was receiving from [APHIA PROGRAM] before transition?
   a. Examples: staff hiring and salaries, commodities, training, funding, support for reporting, patient incentives, etc.
   b. Any support for non-HIV services, like maternal and child health? E.g. antenatal care, immunizations, malaria, etc.

4. Can you explain to me why this facility was selected to no longer receive support from [APHIA PROGRAM]?
   a. When did you find out that the facility would be transitioning?
   b. Who explained the process to you and your colleagues?
   c. How were facility staff members informed about the transfer process?
   d. How were patients informed about the transfer process?

5. What support was provided to this facility in order to prepare it for the transition process?
   a. Were there specific activities that were done to prepare?

b. Were there any assessments done prior to transition to determine facility needs, and if so what were their findings? *(NOTE TO INTERVIEWER: ask for copies of the assessment if possible)*
    i. Who conducted it?
    ii. When?
    iii. How was it scheduled?
    iv. How were the assessment results shared with the facility or with local government?
c. Were meetings held between the facility, [APHIA PROGRAM] and government?
    i. When? How many?
    ii. What was discussed?
d. How did you involve facility staff and patients in planning for the transition?
e. Were there any staff that had been supported by [APHIA PROGRAM] you had to get their salary covered elsewhere? If so, how did you address this?
f. Who provided most of the support to get the facility ready for transition?

6. Can you tell me a little about these transition changes actually occurred?
    a. When did the actual transition process occur?
    b. Was there any confusion in terms of the transition and how it would be implemented? Please explain.
    c. Were all supported activities transferred at the same time?

7. How are HIV/AIDS services at this facility supported now <u>after transition</u>?
    a. What kind of support has the facility received from government? E.g. NACC/NASCOP, central MOH, county health offices, etc.
    b. What kind of support has the facility continued to receive from the [APHIA PROGRAM], if any?
    c. Did the facility receive any funding from [APHIA PROGRAM] after transition? If so, do you know what this covered?
    d. What will this support look like in the next year? Longer-term?

8. What happened to the facility immediately post-transition?
    a. What changes did the facility have to make in the way it operated after transition? E.g. changes to reporting, staffing, etc.

9. How have HIV clinical services at this facility changed post-transition?
    a. Clinical changes:

i. HIV clinical services: HIV testing, treatment, referrals
ii. Community outreach
iii. Pediatric services

b. How has the transition affected the coverage of HIV services?
c. Why have these changes taken place?
   PROBE: changes related to transition or other contextual issue?
d. Did you anticipate any of these changes?
e. Are there any plans to address these changes?
f. Are you aware of any of your patients seeking care from other neighboring facilities, due to the transition?
   i. If so, which services are they seeking elsewhere?
   ii. Which facilities are they going to?
   iii. Why are they no longer seeking services from this facility?

10. How have non-HIV clinical services at this facility changed post-transition?
   a. Clinical changes
      i. Antenatal care
      ii. Family planning
      iii. Malaria
      iv. Tuberculosis
   b. How has the transition affected the coverage of non-HIV services?
   c. Why have these changes taken place?
      PROBE: changes related to transition or other contextual issue?
   d. Did you anticipate any of these changes?
   e. Are there any plans to address these changes?

11. How has the management and organization of this facility changed post-transition?
   a. Health systems changes:
      i. Health workforce
      ii. Community outreach programs and community health workers
      iii. Supply of commodities
      iv. Diagnostic and Lab services
      v. Reporting to DHIS and other information systems

vi. Budgets
vii. Infrastructure (including IT) and maintenance of capital equipment
viii. Any difference between HIV and non-HIV services?

b. Any changes to user fees or the supplies that you ask patients to bring? If so, for what services?
c. Why have these changes taken place?

   PROBE: changes related to transition or other contextual issue?
d. Do you see these as changes for the better or the worse?
e. Did you anticipate any of these changes?
f. Are there any plans to address these changes?

12. How has this facility performed over the longer term since transition?
    a. Has the facility been able to adopt new practices, like test and treat?
        i. If so, was this easy or difficult to do?
        ii. If not, why not? Any plans to do this in the future?
    b. How has the transition affected turnover of staff post transfer?
    c. How has the transition affected staff motivation or performance?

13. How has the county health system responded to the transfer of this facility away from PEPFAR support? Please explain.
    a. How has your relationship with the county health office changed?
    b. Have you collaborated with other facilities? E.g. referrals, commodities, staff.

14. In your view what else should have been done prior to the transfer in order to help with the transition process, which was not done?

15. Is there anything else significant about how the transition process has occurred at this facility that we should know about?

Thank you for your time and contribution

In-Depth Interview Guide #2 (APHIAplus Program)

Project SOAR – Longitudinal Case Studies of PEPFAR Geographic Prioritization

INTERVIEW QUESTIONS

1. Can you tell me a little about your current role, and how familiar you are with [NAME OF SITE]?

INTERVIEWER: If the respondent does not seem at all familiar with the case study facility, then please ask if there is someone else who is more familiar with the facility whom you could talk to.

2. Are you familiar with the recent removal of APHIA Program support and transition of the facility to government support?
    a. Were you involved at all in this process?

INTERVIEWER: If the respondent does not seem at all familiar with transition, then please ask if there is someone else who is more familiar with transition whom you could talk to.

3. Tell me about what types of support your organization was providing before transition?
    a. Examples: staff hiring and salaries, commodities, training, funding, support for reporting, patient incentives, etc.

        PROBE: Facility support, county/district support

    b. Any support for non-HIV services, like maternal and child health? E.g. antenatal care, immunizations, malaria, etc.?

4. Can you explain to me why certain facilities you were working with were selected to transition away from PEPFAR support?
    a. When did you find out that the facility would be transitioning to government?
    b. Who explained the process to you and your colleagues?
    c. How were your colleagues and facility staff members informed about the transfer process?
    d. How were patients informed about the transfer process?

5. What did your organization do internally, as an implementing partner, to prepare for transition?
    a. Examples: meetings, budget reviews, engagement with national level, etc.
    b. Did you face any internal challenges, such loss of funding for particular staff positions, and how did you deal with these?

6. What support was provided to facility/facilities in order to prepare for the transition process?
    a. Were there specific activities that were done to prepare?
    b. Were there any assessments done prior to transition to determine the needs of transitioning facilities, and if so what were their findings? *(NOTE TO INTERVIEWER: ask for copies of the assessment if possible)*
        i. Who conducted it?
        ii. When?
        iii. How was it scheduled?
        iv. How were the assessment results shared with the facility or with local government?
    c. How did you work with government to prepare for transition?
        i. Which part of government did you work with? E.g. county level, central MOH
        ii. Did you hold meetings about the transition process with government? If so, when and how many?
        iii. What was discussed?
    d. How did you involve facility staff and patients in planning for the transition?
    e. Who provided most of the support to get the facility ready for transition?

7. Can you tell me a little about the actual transition process, and how this went?
    a. Were all supported activities transferred at the same time?
    b. How did you sequence the different aspects of the transition process?
    c. What challenges did you experience in terms of the transition and how it was implemented? Please explain.
    d. What agreements were in place between county/district government and your organization regarding support after transition?

8. What kind of relationship, if any, do you now have with this/these transitioned facilities?
    a. What kind of support has the facility continued to receive from you or other implementing partners <u>after transition</u>, if any? (for example, staff hiring and salaries, commodities, training, funding, support for reporting, patient incentives, etc.)
    b. Did the facility receive any funding from your organization? If so, do you know what this covered?

INTERVIEWER: If the respondent no longer has communication with the facility/facilities, skip to question #14

9. What happened to [CASE STUDY FACILITY/IES] immediately post-transition?
    a. What changes did the facility have to make in the way it operated after transition? For example, changes to reporting, staffing, etc.

10. How are activities which were previously supported by [APHIA PROGRAM] supported now?
    a. Who provides this support? E.g. staff hiring and salaries, commodities, training, funding, support for reporting, patient incentives, etc.

        PROBE: Facility support, county/district support

    b. Any support for non-HIV services, like maternal and child health? E.g. antenatal care, immunizations, malaria, etc.?
    c. What kind of support has the facility received from government <u>after transition</u>?
    d. Any additional areas of support that had not been provided before?

11. How have clinical services at the facility changed post-transition?
    a. Clinical changes:
        i. HIV clinical services: HIV testing, treatment, referrals
        ii. Community outreach
        iii. Pediatric services
        iv. Non-HIV services: family planning, malaria, tuberculosis
    b. Why have these changes taken place?

        PROBE: changes related to transition or other contextual issue?

    c. How are these changes perceived by the staff and community?
    d. Did you anticipate any of these changes?
    e. Are there any plans to address these changes?

12. How has the organization and management of the facility itself changed post-transition?
    a. Health systems changes:
        i. Health workforce
        ii. Commodity supply
        iii. Budgets
        iv. Reporting to DHIS
        v. Infrastructure
        vi. Any difference between HIV and non-HIV services?
    b. Why have these changes taken place?

PROBE: changes related to transition or other contextual issue?

c. How are these changes perceived or experienced by the health workers?
d. Did you anticipate any of these changes?
e. Are there any plans to address these changes?

13. How has the facility performed over the longer term since transfer?
    a. Has the facility been able to adopt new practices, like test and treat?
        i. If so, was this easy or difficult to do?
        ii. If not, why not? Any plans to do this in the future?
    b. How has the transition affected turnover of staff post transfer?
    c. How has the transition affected staff motivation or performance?

14. How has the transition affected service coverage?
    a. Why has this happened?
    b. Have there been effects on all the HIV services offered? E.g. PMTCT, ART, prevention, testing, etc.
        i. Why or why not?
    c. How has transition affected non-HIV services, like maternal and child health?
        i. Why has this happened?

15. How has the county/district health system responded to the transfer of these facilities away from PEPFAR support? Please explain.
    a. How has your relationship with the county/district health office changed?
    b. How has your relationship with PEPFAR changed?
    c. [IF PARTNER STILL ENGAGES WITH TRANSITIONED FACILITIES]: How has your relationship with the facility changed?

16. In your view what else should have been done prior to the transfer in order to help with the transition process, which was not done?

17. How does what you have observed at this facility compared to other transitioned facilities that you have supported?

18. Is there anything else significant about how the transition process has occurred at this facility that we should know about?

Thank you for your time and contribution

In-Depth Interview Guide #3 (County Health Office)

Project SOAR – Longitudinal Case Studies of PEPFAR Geographic Prioritization

INTERVIEW QUESTIONS

1. Can you tell me a little about your current role, and how familiar you are with this facility (NAME OF SITE)?

INTERVIEWER: If the respondent does not seem at all familiar with the case study facility, then please ask if there is someone else who is more familiar with the facility whom you could talk to.

2. Are you familiar with the recent removal of APHIA Program support and transition of the facility to government support?

   PROBE: Have you had any recent changes in how [APHIA PROGRAM] has supported the facility?

   a. Were you involved at all in this transition process at this facility?

INTERVIEWER: If the respondent does not seem at all familiar with transition, then please ask if there is someone else who is more familiar with transition whom you could talk to.

3. Can you explain to me why this county was selected to transition away from [PEPFAR/APHIA PROGRAM] support?
   a. When did you find out that this county would be transitioning to government?
   b. Who explained the process to you and your colleagues?
   c. Were facilities informed officially of the transfer process? If so, by whom?
   d. How were facility staff members informed about the transfer process?
   e. How were patients informed about the transfer process?

4. What did the county health office do to prepare for transition?
   a. Examples: meetings, budget reviews, engagement with national level, etc.
   b. Was there any preparation done by other county entities?

5. What support was provided by <u>your team</u> to [NAME OF SITE] in order to prepare it for the transition process?
   a. Were there specific activities that were done to prepare?
   b. Were there any assessments done prior to transition to determine facility needs, and if so what were their findings? *(NOTE TO INTERVIEWER: ask for copies of the assessment if possible)*

i. Who conducted it?

ii. When?

iii. How was it scheduled?

iv. How were the assessment results shared with the facility?

c. How did you work with [APHIA PROGRAM] and the facilities to prepare for transition?

i. Did you hold meetings about the transition process with government and the facility? If so, when and how many?

ii. What was discussed?

d. How did you involve facility staff and patients in planning for the transition?

e. Who provided most of the support to get facilities ready for transition?

6. Can you tell me a little about the actual transfer process, and how this went?

a. Was there any confusion in terms of the transition and how it would be implemented? Please explain.

b. Were all supported activities transferred at the same time?

7. Can you tell me about what types of support [NAME OF SITE] was receiving from [APHIA PROGRAM] before transition?

a. Examples: staff hiring and salaries, commodities, training, funding, support for reporting, patient incentives, etc.

b. Any support for non-HIV services, like maternal and child health? E.g. antenatal care, immunizations, malaria, etc.?

8. How is [NAME OF SITE] supported now <u>after transition</u>?

a. What kind of support has [NAME OF SITE] received from government <u>after transition</u>? E.g. NACC/NASCOP, central MOH, county government.

b. What kind of support have the local government or facility continued to receive from the [APHIA PROGRAM] <u>after transition</u>, if any?

c. Does this support vary by type or size of the facility?

d. What agreements were in place between county government and [APHIA PROGRAM] regarding support after transition?

9. How have clinical services changed at [NAME OF SITE] post-transition?

a. Clinical changes:

i. HIV clinical services: HIV testing, treatment, referrals

ii. Community outreach

   iii. Pediatric services
   iv. Non-HIV services: family planning, malaria, tuberculosis

b. How has the transition affected service coverage?
c. Why have these changes taken place?

   PROBE: changes related to transition or other contextual issue?

d. Did you anticipate any of these changes?
e. Are there any plans to address these changes?
f. Are you aware of any shifts in patient patterns of care seeking – for example do you think patients are shifting from transition facilities to those that continue to receive support from [PEPFAR / APHIA PROGRAM]?

10. How has the organization and management of [NAME OF SITE] changed post-transition?
    a. Health systems changes:
       i. Health workforce
       ii. Commodity supply
       iii. Budgets
       iv. Reporting to DHIS
       v. Infrastructure
       vi. Any difference between HIV and non-HIV services?
    b. Why have these changes taken place?

       PROBE: changes related to transition or other contextual issue?

    c. How do you perceive these changes, do you think they are for the better or for the worse?
    d. Did you anticipate any of these changes?
    e. Are there any plans to address these changes?

11. How has [NAME OF SITE] performed over the longer term since transfer?
    a. Has the facility been able to adopt new practices, like test and treat?
       i. If so, was this easy or difficult to do?
       ii. If not, why not? Any plans to do this in the future?
    b. How has the transition affected turnover of staff post transfer?
    c. How has the transition affected staff motivation or performance?

12. Has service coverage changed? If so, how?
    a. Why has this happened?

  b. Have there been effects on all the HIV services offered? E.g. PMTCT, ART, prevention, testing, etc.
     i. Why or why not?
  c. How have non-HIV services, like maternal and child health, changed?
     i. Why has this happened?

13. What happened to <u>your team</u> immediately post-transition?
    a. What changes did you have to make in the way you operated after transition? For example, changes to reporting, staffing, etc.
    b. What changes did the facility have to make in the way it operated after transition? E.g. changes to reporting, staffing, etc.

14. How has the county health system responded to the transfer of [NAME OF SITE] away from PEPFAR support? Please explain.
    a. How has your relationship with the facilities changed?
    b. How has your relationship with the national level changed?
    c. Have you collaborated with other county governments? E.g. referrals, commodities, staff.
    d. Describe the major challenges you were facing during this period.

15. Can you tell me about what types of support <u>your team</u> is currently receiving from [APHIA PROGRAM]?
    a. Examples: facilitation for meetings, supplies, transport or fuel for transport, staff hiring, training, support for reporting etc.
    b. Was this support targeted for a particular health area (e.g. HIV service management and planning, maternal and child health)?
    c. How is this support different from the support [APHIA PROGRAM] provided to your county before transition? Why?
16. In your view what else should have been done prior to the transfer in order to help with the transition process, which was not done?

17. How does what you have observed at [NAME OF SITE] compare to other transitioned facilities that you have supported?

18. Is there anything else significant about how the transition process has occurred at [NAME OF SITE] that we should know about?

Thank you for your time and contribution

Focus Group Guide #4 (Patients)

Project SOAR – Longitudinal Case Studies of PEPFAR Geographic Prioritization

QUESTIONS

1. Please can you introduce yourself and tell me how long have you been attending this facility?

INTERVIEWER: Please ask each individual their name, age and how far they live from the facility.

2. Have there been any changes in the services that this facility provides since September, 2016?
    a. Do you see any changes in the types of services that the facility provides? If so, please explain.

       PROBE: HIV and non-HIV services, like maternal and child health
    b. Do you see any changes in the quality of services that the facility provides? If so, please explain.

       PROBE: HIV and non-HIV services, like maternal and child health
    c. Was there any interruption in services?

3. What kind of changes, if any, have you noticed in the way the facility operates since September 2016?
    a. Have you noticed, any changes in staffing or the way services were provided? Please explain.
    b. Have you seen any changes in:
        i. Staff motivation and turnover
        ii. Drug Availability
        iii. Lab services
    c. Do you think the staff are as motivated as they were before?
    d. Has there been much turnover of staff?

4. How do you feel about the changes at the facility recently?
    a. Would you consider switching to another facility to receive your services? Why/why not?
        i. If so, where would you go? Why?
        ii. If so, do you think it would be harder or easier to get the care you want? Why/why not?

5. Are you aware that this facility used to receive support from the APHIA Program, but is no longer getting that support?
    a. Did anyone inform you about the transition process?
        i. If so, who? When? How?
    b. Was it clear to you what was going on in terms of what was happening and when?

6. Were you aware of any steps that were taken to get the facility ready for the removal of support from the APHIA Program?
    a. Were there any unexpected problems in transition, or would you say it was a relatively smooth process?

7. In your view was there anything that really should have been done prior to the removal of APHIA Program support in order to help with the transition process, which was not done?

8. Do you think there is anything else significant about how the transition process has occurred at this facility that we should know about?

Many thanks for your help and time.
