# Supplementary material for: Effects of transition on HIV and non-HIV services and health systems in Kenya: a mixed methods evaluation of donor transition
Source: BMC Health Serv Res. 2021 May 13;21:457. doi: 10.1186/s12913-021-06451-y (PMC8117613; doi:10.1186/s12913-021-06451-y)
Supplement: Supplementary file 4 — Additional file 4. [file 12913_2021_6451_MOESM4_ESM.zip › SOAR_Comp3_IntGuides Maint R1R2.pdf]

In-Depth Interview Guide #1 (Facility In-Charge)

Project SOAR – Longitudinal Case Studies of PEPFAR Geographic Prioritization

INTERVIEW QUESTIONS

1. Can you tell me a little about your current role, and how familiar you are with this facility?

INTERVIEWER: If the respondent does not seem at all familiar with the case study facility, then please ask if there is someone else who is more familiar with the facility whom you could talk to.

2. Are you familiar with the recent geographic prioritization of PEPFAR support in Kenya?

   PROBE: Have you had any recent changes in how [APHIA PROGRAM] has supported the facility?

INTERVIEWER: If respondent does not understand Geographic Prioritization, use the following description:

"PEPFAR, the US government program that funds APHIAPlus Kamili, has decided to prioritize their support in different parts of Kenya based on the HIV burden that is found in each county. They will be focusing its support and funding in counties with medium and high burden, and reducing support in counties with low burden."

3. Are you aware that this facility has been selected to have its PEPFAR support maintained during the Geographical Prioritization process?

   ➔ If YES,

      i. Can you explain why this decision was made?

      ii. When did you find out that the facility would maintain its support from [APHIA PROGRAM]?

      iii. Who explained this decision to you and your colleagues?

   ➔ If NO, go to #4

4. Can you tell me about what types of support your facility receives from [APHIA PROGRAM] for HIV services?

   a. Examples: staff hiring and salaries, commodities, training, funding, support for reporting, patient incentives, etc.

   b. Any support for non-HIV services, like maternal and child health? E.g. antenatal care, immunizations, malaria, etc.

   c. Is this different than before [INSERT TIME OF TRANSITION HERE]? If so, how is it different?

   d. Do you expect any of this to change?

5. Who else supports your facility? E.g. county government, central MOH, NACC/NASCOP, etc.
    a. What kind of support does the facility receive from others?
       PROBE: ask this for every other organization mentioned (e.g. NASCOP, county, etc)
    b. Any additional areas of support that had not been provided before?
    c. What will this support look like in the next year? Longer-term?

6. How have clinical services at the facility changed since [INSERT TIME FO TRANSITION HERE]?
    a. Clinical changes:
        i. HIV clinical services: HIV testing, treatment, referrals
        ii. Community outreach
        iii. Pediatric services
    b. Why have these changes taken place?
       PROBE: changes related to Geographic Prioritization or other contextual issue?
    c. Did you anticipate any of these changes?
    d. Are there any plans to address these changes?

7. How have non-HIV clinical services at this facility changed since [INSERT TIME FO TRANSITION HERE]?
    a. Clinical changes
        i. Antenatal care
        ii. Family planning
        iii. Malaria
        iv. Tuberculosis
    b. Why have these changes taken place?
       PROBE: changes related to transition or other contextual issue?
    c. Did you anticipate any of these changes?
    d. Are there any plans to address these changes?

8. How has the management and organization of the facility changed since [INSERT TIME FO TRANSITION HERE]?
    a. Health systems changes:
        i. Health workforce
        ii. Community outreach programs and community health workers
        iii. Supply of commodities

iv. Diagnostic and Lab services
v. Reporting to DHIS and other information systems
vi. Budgets
vii. Infrastructure (including IT) and maintenance of capital equipment
viii. Any difference between HIV and non-HIV services?

b. Any changes to user fees or the supplies that you ask patients to bring? If so, for what services?
c. Why have these changes taken place?

   PROBE: changes related to Geographic Prioritization or other contextual issue?
d. Do you see these as changes for the better or the worse?
e. Did you anticipate any of these changes?
f. Are there any plans to address these changes?

9. How has the facility performed over the longer term since [INSERT TIME FO TRANSITION HERE]?
   a. Has the facility been able to adopt new practices, like test and treat?
      i. If so, was this easy or difficult to do?
      ii. If not, why not? Any plans to do this in the future?
   b. How has turnover of staff changed?
   c. How has staff motivation or performance changed?

10. How has the county health system responded to recent changes in support from [APHIA Program]? Please explain.
    a. How has your relationship with the county health office changed?
    b. Have you collaborated with other facilities? E.g. referrals, commodities, staff.

11. In your view what else should have been done in order to help with the recent changes, which was not done?

12. Is there anything else significant about how the Geographic Prioritization process has occurred at this facility that we should know about?

Thank you for your time and contribution

In-Depth Interview Guide #2 (APHIAplus Program)

Project SOAR – Longitudinal Case Studies of PEPFAR Geographic Prioritization

INTERVIEW QUESTIONS

1. Can you tell me a little about your current role, and how familiar you are with [NAME OF SITE]?

INTERVIEWER: If the respondent does not seem at all familiar with the case study facility, then please ask if there is someone else who is more familiar with the facility whom you could talk to.

2. Are you familiar with the recent geographic prioritization of PEPFAR support in Kenya?
    a. Were you involved at all in this process on behalf of [APHIA PROGRAM]?

INTERVIEWER: If the respondent does not seem at all familiar with Geographic Prioritization, then please ask if there is someone else who is more familiar whom you could talk to.

3. Tell me about what types of support your organization was providing before [INSERT TIME OF TRANSITION HERE]?
    a. Examples: staff hiring and salaries, commodities, training, funding, support for reporting, patient incentives, etc.

        PROBE: Facility support, county/district support

    b. Any support for non-HIV services, like maternal and child health? E.g. antenatal care, immunizations, malaria, etc.?

4. How are these activities supported now? By whom?
    a. Examples: staff hiring and salaries, commodities, training, funding, support for reporting, patient incentives, etc.

        PROBE: Facility support, county/district support

    b. Any support for non-HIV services, like maternal and child health? E.g. antenatal care, immunizations, malaria, etc.?
    c. Any additional areas of support that had not been provided before?

5. IF ANY CHANGES REPORTED IN #4: What did your organization, as an implementing partner, do to prepare for these changes?
    a. Examples: meetings, budget reviews, engagement with national level, etc.

6. How have clinical services at [NAME OF SITE] changed <u>since</u> [INSERT TIME OF TRANSITION HERE]?
    a. Clinical changes:
        i. HIV clinical services: HIV testing, treatment, referrals
        ii. Community outreach
        iii. Pediatric services
        iv. Non-HIV services: family planning, malaria, tuberculosis
    b. Why have these changes taken place?
       PROBE: changes related to PEPFAR geographic prioritization or other contextual issue?
    c. How are these changes perceived by the staff and community?
    d. Did you anticipate any of these changes?
    e. Are there any plans to address these changes?

7. How has the organization and management of [NAME OF SITE] changed <u>since</u> [INSERT TIME OF TRANSITION HERE]?
    a. Health systems changes:
        i. Health workforce
        ii. Commodity supply
        iii. Budgets
        iv. Reporting to DHIS
        v. Infrastructure
        vi. Any difference between HIV and non-HIV services?
    b. Why have these changes taken place?
       PROBE: changes related to PEPFAR geographic prioritization or other contextual issue?
    c. How are these changes perceived or experienced by the health workers?
    d. Did you anticipate any of these changes?
    e. Are there any plans to address these changes?

8. How has [NAME OF SITE] performed over the longer term <u>since</u> [INSERT TIME OF TRANSITION HERE]?
    a. Has the facility been able to adopt new practices, like test and treat?
        i. If so, was this easy or difficult to do?
        ii. If not, why not? Any plans to do this in the future?
    b. How has turnover of staff changed?
    c. How has staff motivation or performance changed?

9. Has service coverage changed since [INSERT TIME OF TRANSITION HERE]? If so, how?
    a. Why has this happened?
    b. Have there been effects on all the HIV services offered? E.g. PMTCT, ART, prevention, testing, etc.
        i. Why or why not?
    c. How have non-HIV services, like maternal and child health, changed?
        i. Why has this happened?

10. How has your relationship with the county health system changed in response to the geographic reprioritization and any recent changes in PEPFAR support? Please explain.
    a. How has your relationship with the county health office changed?
    b. How has your relationship with PEPFAR changed?
    c. How has your relationship with [NAME OF SITE] changed?

11. In your view what else should have been done in order to facilitate with the geographic reprioritization process, which was not done?

12. Broadly speaking, what is your general impression about the geographic prioritization process, and how this went?
    a. What challenges did facilities, county health teams, and implementing partners experience in terms of the geographic prioritization and how it was implemented? Please explain.

13. Is there anything else significant about how the geographic prioritization process has occurred at [NAME OF SITE] that we should know about?

Thank you for your time and contribution

In-Depth Interview Guide #3 (County Health Office)

Project SOAR – Longitudinal Case Studies of PEPFAR Geographic Prioritization

INTERVIEW QUESTIONS

1. Can you tell me a little about your current role, and how familiar you are with the [NAME OF SITE] facility?

INTERVIEWER: If the respondent does not seem at all familiar with the case study facility, then please ask if there is someone else who is more familiar with the facility whom you could talk to.

2. Are you familiar with the recent geographic prioritization of PEPFAR support in Kenya?

   PROBE: Have you had any recent changes in how [APHIA PROGRAM] has supported [NAME OF SITE] or your county team?

   a. Were you involved at all in any discussions about Geographic prioritization at [NAME OF SITE] or in the county more broadly?

INTERVIEWER: If respondent does not understand Geographic Prioritization, use the following description:

"PEPFAR, the US government program that funds APHIAPlus Kamili, has decided to prioritize their support in different parts of Kenya based on the HIV burden that is found in each county. They will be focusing its support and funding in counties with medium and high burden, and reducing support in counties with low burden."

3. Can you tell me about what types of support [NAME OF SITE] receives from [APHIA PROGRAM] for HIV services?

   a. Examples: staff hiring and salaries, commodities, training, funding, support for reporting, patient incentives, etc.
   b. Any support for non-HIV services, like maternal and child health? E.g. antenatal care, immunizations, malaria, etc.?
   c. What agreements are in place between county government and [APHIA PROGRAM] regarding support?
   d. Who else supports [NAME OF SITE]? E.g. county government, central MOH, NACC/NASCOP, etc.
   e. Do you expect any of this to change?
   f. Is this different than before [INSERT TIME OF TRANSITION HERE]? If so, how is it different?

4. How have clinical services changed at [NAME OF SITE] <u>since</u> [INSERT TIME OF TRANSITION HERE]?

a. Clinical changes:

   i. HIV clinical services: HIV testing, treatment, referrals

   ii. Community outreach

   iii. Pediatric services

   iv. Non-HIV services: family planning, malaria, tuberculosis

b. Why have these changes taken place?

   PROBE: changes related to Geographic Prioritization or other contextual issue?

c. Did you anticipate any of these changes?

d. Are there any plans to address these changes?

e. Are you aware of any shifts in patient patterns of care seeking – for example do you think patients are shifting from transition facilities to those that continue to receive support from [APHIA PROGRAM]?

5. How has the organization and management of [NAME OF SITE] changed since [INSERT TIME OF TRANSITION HERE]?

   a. Health systems changes:

      i. Health workforce

      ii. Commodity supply

      iii. Budgets

      iv. Reporting to DHIS

      v. Infrastructure

      vi. Any difference between HIV and non-HIV services?

   b. Why have these changes taken place?

      PROBE: changes related to Geographic Prioritization or other contextual issue?

   c. How do you perceive these changes, do you think they are for the better or for the worse?

   d. Did you anticipate any of these changes?

   e. Are there any plans to address these changes?

6. How has [NAME OF SITE] performed <u>since</u> [INSERT TIME OF TRANSITION HERE]?

   a. Has the facility been able to adopt new practices, like test and treat?

      i. If so, was this easy or difficult to do?

      ii. If not, why not? Any plans to do this in the future?

   b. How has turnover of staff changed?

   c. How has staff motivation or performance changed?

7. Has service coverage changed? If so, how?
    a. Why has this happened?
    b. Have there been effects on all the HIV services offered? E.g. PMTCT, ART, prevention, testing, etc.
        i. Why or why not?
    c. How have non-HIV services, like maternal and child health, changed?
        i. Why has this happened?

8. Can you tell me about what types of support <u>your team</u> is currently receiving from [APHIA PROGRAM]?
    a. Examples: facilitation for meetings, supplies, transport or fuel for transport, staff hiring, training, support for reporting etc.
    b. Was this support targeted for a particular health area (e.g. HIV service management and planning, maternal and child health)?
    c. Is this different from the support your team received before [INSERT TIME OF TRANSITION HERE]? If so, how is it different?

9. How has the county health system responded to these recent changes in [APHIA PROGRAM] support? Please explain.
    a. How has your relationship with the facilities changed?
    b. How has your relationship with the national level changed?
    c. Have you collaborated with other county governments? E.g. referrals, commodities, staff.

10. How does what you have observed at [NAME OF SITE] compare to other facilities in your county?

11. In your view what else should have been done in order to facilitate the geographic reprioritization process, which was not done?

12. Is there anything else significant about how the geographic prioritization process has occurred at [NAME OF SITE] that we should know about?

Thank you for your time and contribution

Focus Group Guide #4 (Patients)

Project SOAR – Longitudinal Case Studies of PEPFAR Geographic Prioritization

QUESTIONS

1. Please can you introduce yourself and tell me how long have you been attending this facility?

INTERVIEWER: Please ask each individual their name, age and how far they live from the facility.

2. Have there been any changes in the services this facility provides since [INSERT TIME OF TRANSITION HERE]?
    a. Do you see any changes in the <u>types of services</u> that the facility provides? If so, please explain.

       PROBE: HIV and non-HIV services, like maternal and child health
    b. Do you see any changes in the <u>quality of services</u> that the facility provides? If so, please explain.

       PROBE: HIV and non-HIV services, like maternal and child health
    c. Was there any interruption in services?

3. What kind of changes, if any, have you noticed in the way the facility operates since September 2016?
    a. Have you noticed, any changes in staffing or the way services were provided? Please explain.
    b. Have you seen any changes in:
        i. Staff motivation and turnover
        ii. Drug Availability
        iii. Lab services
    c. Do you think the staff are as motivated as they were before?
    d. Has there been much turnover of staff?

4. IF YES TO #2 OR #3: How do you feel about the changes at the facility recently?
    a. Would you consider switching to another facility to receive your services? Why/why not?

i. If so, where would you go? Why?

ii. If so, do you think it would be harder or easier to get the care you want? Why/why not?

5. Do you think there is anything else significant about recent changes taking place at this facility that we should know about?

Many thanks for your help and time.
