# Supplementary material for: Effects of transition on HIV and non-HIV services and health systems in Kenya: a mixed methods evaluation of donor transition
Source: BMC Health Serv Res. 2021 May 13;21:457. doi: 10.1186/s12913-021-06451-y (PMC8117613; doi:10.1186/s12913-021-06451-y)
Supplement: Supplementary file 4 — Additional file 4. [file 12913_2021_6451_MOESM4_ESM.zip › SOAR_Comp3_IntGuides R2.pdf]

Project SOAR – Longitudinal Case Studies of PEPFAR Geographic Prioritization

SEMI-STRUCTURED INTERVIEW GUIDE – PEPFAR IMPLEMENTING PARTNER – CENTRAL SUPPORT

(ROUND 2)

INTRODUCTION

Thank you for agreeing to meet us.

We are conducting an assessment of PEPFAR's geographic prioritization process; that is, the process through which sites have transitioned from PEPFAR support to central support. We are interested in the processes that took place before transition to prepare and after transition under central support. Our goal is to provide practical information to local and national government, PEPFAR and other partners about how the transition process took place and whether it has affected how services are delivered.

As part of the overall evaluation, we are conducting case studies of facilities that are transitioning from PEPFAR support and some that are being sustained. We are studying [NAME OF SITE] and would like to ask you some questions regarding this particular facility, but we also want you to reflect on the transition process that all facilities are going through. We conducted a first round of interviews earlier this year, and we have returned to understand how things have changed since then.

Name of Organization ____________________

Your name ____________________

Designation ____________________

Work Area ____________________

Postal address ____________________

Telephone ____________________

E-mail address ____________________

OBTAIN INFORMED CONSENT

*NOTE TO INTERVIEWER: This is a guide to the interview. You should cover **all the main numbered questions** in this interview form. You should use the probes selectively, according to the type of knowledge that the respondent conveys, and what you have already found out from documents and other interviews.*

INTERVIEW QUESTIONS

***INTERVIEWER**: If respondent was interviewed before and position has not changed, skip Q1 and Q2. If respondent was not interviewed before or if role has changed, ask Q1 and Q2*

1. **New respondents only:** Can you tell me a little about your current role, and how familiar you are with [NAME OF SITE]?

***INTERVIEWER**: If the respondent does not seem at all familiar with the case study facility, then please ask if there is someone else who is more familiar with the facility whom you could talk to.*

2. **New respondents only:** Are you familiar with the recent PEPFAR policy through which certain districts and facilities transition away from [PEPFAR OR IMPLEMENTING PARTNER] support?
    a. Were you involved at all in this transition process?

***INTERVIEWER**: If the respondent does not seem at all familiar with transition, then please ask if there is someone else who is more familiar with transition whom you could talk to.*

3. What is the current status of [NAME OF SITE] in regards to PEPFAR's Geographic Prioritization? Is this facility still receiving direct PEPFAR support?

***INTERVIEWER** Share the status reported in previous data collection rounds for this facility.*

    a. *IF THE RESPONSE IS DIFFERENT FROM STATUS REPORTED IN ROUND 1:* When did this change happen? Why?
    b. Who, if anyone explained the process ad rationale to you?

4. What kind of relationship, if any, have you had with [NAME OF SITE] since May 2017?

Support for Services

INTERVIEWER: Now I'm going to ask you about how support for the facility has changed since PEPFAR's Geographic Prioritization.

5. What kind of support, if any, has the facility received from you since May 2017? (E.g. staff hiring and salaries, commodities, training, funding, support for reporting, patient incentives, etc.)

    PROBE: Which IP providing which support, frequency, etc.

    a. Has the support given changed since then?
    b. Has the facility lost any support from you? Why did they lose support?
    c. Any additional areas of support that had not been provided before?

6. How are non-HIV services that used to be offered through the APHIAs provided now?
    a. Are non-HIV services also prioritized geographically?

i. If so, which services are prioritized and why?

ii. Is there any support to Central Support counties at all? If so, what?

INTERVIEWER*: If the respondent no longer has communication with the facility/facilities, skip to question #11*

7. How are HIV activities which were previously supported by your organization supported now?
   a. Who provides this support? For example for staff hiring and salaries, commodities, training, funding, support for reporting, patient incentives, etc.

      PROBE: Facility support, county support

   b. What kind of support has the facility received from the county since May 2017? From the MOH?
   c. Any additional areas of support that had not been provided before?
   d. How is this arrangement different from how activities were supported before May 2017?
   e. What agreements were in place between county government and [IMPLEMENTING PARTNER] regarding support since May 2017?

8. Who else currently works with or support [NAME OF SITE] that you are aware of? E.g. county health offices, national AIDS agencies, PEPFAR supported partners, non-PEPFAR supported partners, civil society, etc.
   a. What support do they provide?

   PROBE: HIV service delivery support, HIV outreach, MCH or non-HIV support

   b. When did this support start? Is it ongoing?
      i. IF SUPPORT IS NEW: What prompted the start of this support?
      ii. Are you aware of when support is supposed to end? Why?
   c. Has your organization collaborated with these organizations to provide support to [NAME OF SITE]?

Effects of Geographic Prioritization

INTERVIEWER*:* Now I would like to ask you a series of questions about the effects of PEPFAR's geographic prioritization at [NAME OF SITE].

9. How have clinical services at [NAME OF SITE] changed since May 2017?
   a. Clinical changes:
      i. HIV clinical services: HIV testing, treatment, referrals
         1. ***Is [NAME OF SITE] providing CD4 tests?
      ii. Community outreach

iii. Pediatric services

iv. Non-HIV services: family planning, malaria, tuberculosis

1. ***Is [NAME OF SITE] providing hematology tests?

b. Why have these changes taken place?

PROBE: changes related to Geographic Prioritization or other contextual issue?

c. How do you perceive these changes: do you think they are for the better or the worse?

d. How are these changes perceived by the staff and community?

e. Did you anticipate any of these changes?

f. Are there any plans to address these changes?

g. Are you aware of any shifts in patient patterns of care seeking – for example do you think patients are shifting from transition facilities to those that continue to receive Implementing partner support?

i. ***MARSABIT ONLY: Are you aware of any information being spread on the radio and local news of HIV services ending at Tumaini? If so, who was providing this information? What happened to patient care seeking behavior?

10. How has the organization and management of the facility itself changed since May 2017?

a. Health systems changes:

i. Health workforce- has [NAME OF SITE] lost or gained staff? Any changes to motivation or performance?

ii. Commodity supply – have there been problems with drug availability or costs of drugs at [NAME OF SITE]?

1. ***Are you aware of [NAME OF SITE] charging for any drugs? If so, which drugs?

iii. Lab services – are you aware of any problems related to the array of tests offered to clients? The turnaround time for obtaining test results?

1. ***Are you aware of [NAME OF SITE] charging for any tests? If so, which tests?

2. ***Has [NAME OF SITE] experienced difficulties with transporting lab samples?

a. For Viral Load?

b. For Early Infant Diagnosis (EID)?

c. For Gene Expert?

iv. Budgets – any notable changes to budgets and expenditures for [NAME OF SITE]?

v. Reporting to DHIS – has [NAME OF SITE] been able to maintain regular reporting to DHIS?

vi. Infrastructure

vii. Any difference between HIV and non-HIV services?

b. Why have these changes taken place?

   PROBE: changes related to Geographic Prioritization or other contextual issue?

c. How do you perceive these changes: do you think they are for the better or the worse?

d. How are these changes perceived or experienced by the health workers?

e. Did you anticipate any of these changes?

f. Are there any plans to address these changes?

   i. If so, who will be responsible? E.g. county leadership, PEPFAR implementing partners, national government, other donors, etc.

11. How has [NAME OF SITE] performed overall since May 2017?

    a. Has [NAME OF SITE] been able to adopt new practices, like test and treat?

       i. If so, was this easy or difficult to do?

       ii. If not, why not? Any plans to do this in the future?

12. How has service coverage changed since May 2017?

    a. Why has this happened?

    PROBE: changes related to Geographic Prioritization or other contextual issue?

    b. Have there been effects on the HIV services offered? E.g. PMTCT, ART, prevention, testing, etc.

       i. Why or why not?

    c. Have there been effects on non-HIV services, like maternal and child health?

       i. Why has this happened?

       ii. How has this support replaced the support [your organization / IP NAME] was providing to the facility?

    d. What agreements between county government and your organization have been put in place or retained since transition?

13. What support has been provided to [NAME OF SITE] in order to address the effects of Geographic Prioritization?

    a. Are there specific activities that have been done or are ongoing? E.g. planning meetings, trainings, etc.

    b. Have you or other implementing partners helped facilities or the overall district develop work plans to cope with the transition and/or loss of support?

    c. Have there been any assessments to monitor how the facility is faring after transition?

        i. If so, who conducted it? When?
        ii. How are the results from the assessment being used? By whom?
    d. Who has provided the support to the facility since transition?

14. How does what you have observed at [NAME OF SITE] compare to other transitioned facilities that you have supported?

15. What kind of relationship, if any, have you had with county health office since May 2017?
    a. What kind of support has the county received from you or other implementing partners since May 2017, if any? (E.g. staff hiring and salaries, commodities, training, funding, support for reporting, patient incentives, etc.)
        i. ***Have you supported the county with work planning and budgeting?

    PROBE: Which IP providing which support, frequency, etc.

    b. Did the county receive any funding from your organization? If so, do you know what this covered?
        i. Do they continue to receive funding from your organization?

INTERVIEWER*: If the respondent no longer has communication with the county/district, skip to question #17*

16. How has the county/district health system responded to the transfer of these facilities away from PEPFAR support? Please explain.
    a. How has your relationship with the county/district health office changed?

17. How has your organization changed how it operates since May 2017?
    a. Did you face any internal challenges, such loss of funding for particular staff positions, and how did you deal with these?
    b. How has your relationship with PEPFAR changed?

18. How did the support for [NAME OF SITE] and [COUNTY NAME] change when the APHIA contracts ended?

    PROBE: HIV treatment, prevention, outreach, testing, above-site, workplanning, etc.

    a. What counties does your current program cover now?
        i. How similar or different is your catchment area than the catchment areas under APHIA?
    b. How did the process of switching between implementing partners and/or contracts go?

i. Where there any breaks in support to counties? If so, what happened?

c. How did counties and facilities react to the switch in programs?

19. Broadly speaking, what is your general impression about the Geographic Prioritization process, and how this went?
    a. How has your opinion changed since May 2017?
    b. What challenges did facilities, local government teams, and implementing partners experience in terms of the Geographic Prioritization and how it was implemented? Please explain.
    c. What else should have been done in order to facilitate the Geographic Prioritization process, which was not done?
    d. How do you think the relationship between county and PEPFAR IP support will change in the next six months? In the next year?
    e. What do you think will happen when your project ends?

20. In your view what else should have been done prior to the transfer in order to help with the Geographic Prioritization process, which was not done?

21. Is there anything else significant about how the transition process has occurred at this facility that we should know about?

Thank you for your time and contribution

Project SOAR – Longitudinal Case Studies of PEPFAR Geographic Prioritization

<u>SEMI-STRUCTURED INTERVIEW GUIDE – FACILITY IN-CHARGE (ROUND 2)</u>

INTRODUCTION

Thank you for agreeing to meet us.

We are conducting an assessment of PEPFAR's Geographic Prioritization process; that is, the process through which sites have transitioned from PEPFAR support to central support. Transfer of support has happened at some facilities in Kenya but not in others. We are interested in how support to facilities has changed and the effects those changes have had on services.

Our goal is to provide practical information to local and national government, PEPFAR and other partners about how the transition process took place and whether it has affected how services are delivered. As part of the overall evaluation, we are conducting case studies with a number of specific facilities. We conducted a first round of interviews earlier this year, and we have returned to understand how things have changed since then.

Name of Organization ______________________

Your name ______________________

Designation ______________________

Work Area ______________________

Postal address ______________________

Telephone ______________________

E-mail address ______________________

OBTAIN INFORMED CONSENT

NOTE TO INTERVIEWER: This is a guide to the interview. You should cover ***all the main numbered questions*** *in this interview form. You should use the probes selectively, according to the type of knowledge that the respondent conveys, and what you have already found out from documents and other interviews.*

INTERVIEW QUESTIONS

***INTERVIEWER**: If respondent was interviewed before and position has not changed, skip Q1 and Q2. If respondent was not interviewed before or if role has changed, ask Q1 and Q2*

1. **New respondents only:** Can you tell me a little about your current role, and how familiar you are with [NAME OF SITE]?

INTERVIEWER: If the respondent does not seem at all familiar with the case study facility, then please ask if there is someone else who is more familiar with the facility whom you could talk to.

2. **New respondents only:** Are you familiar with the recent transition away from [PEPFAR OR IMPLEMENTING PARTNER] support?
   a. Were you involved at all in this transition process?

***INTERVIEWER**: FOR CENTRAL SUPPORT facility: If the respondent does not seem at all familiar with transition, then please ask if there is someone else who is more familiar with transition whom you could talk to. If there is no one more familiar with the transition, continue interview.*

3. What is the current status of this facility in regards to PEPFAR's Geographic Prioritization or transition? Is this facility still receiving direct PEPFAR support?

   ***INTERVIEWER:** Share the status reported in previous data collection rounds for this facility.*

   a. *IF THE RESPONSE IS DIFFERENT FROM STATUS REPORTED IN ROUND 1:* When did this change happen? Why?
      i. Who, if anyone, explained the process to you?

Support for Facility

INTERVIEWER: Now I'm going to ask you about how support for the facility has changed since PEPFAR's Geographic Prioritization.

4. How have facility services been supported since May 2017? E.g. facilitation for meetings, supplies, transport or fuel for transport, staff hiring, training, support for reporting etc.
   a. What kind of support has the facility continued to receive from the [IMPLEMENTING PARTNER], if any?
      i. Please describe all of the current projects and/or partners that have been supporting your facility since May 2017.

         PROBE: PEPFAR implementing partners/projects, non-PEPFAR implementing partners/projects

      ii. When did these projects/partners start supporting your facility?
   b. Has the facility received any funding from any partners since May 2017?
      i. If so, which partner provided the funds? What were they meant to cover?

c. What kind of support has the facility received from government? E.g. [NATIONAL AIDS CONTROL ORGANIZATIONS], central MOH, county health offices, etc.

    i. ***What specific support does the county health office provide you with? E.g. supervision, lab transport, reporting, funding, workplanning, etc.

d. Any support for non-HIV services, like maternal and child health? E.g. antenatal care, immunizations, malaria, etc.

e. What will this support look like in the next year? Longer-term?

5. Please tell me about all of the projects/implementing partners that <u>have stopped supporting</u> the facility since May 2017.

   PROBE: PEPFAR implementing partners/projects, non-PEPFAR implementing partners/projects

   a. When did they stop their support?
   b. Why did they stop their support?
   c. What support were they providing?
   d. What happened to that support after they left?

6. How much of this support is new since May 2017?

   PROBE: new partners, new activities, changes between partners and government, etc.

   a. What brought about this new support? E.g. newly identified need, new contracts issued, etc.
   b. Do you anticipate any new partners to start providing support within the next 6 months?

7. FOR CENTRAL SUPPORT ONLY: What support did this facility receive in order to manage the effects of transition since May 2017?

   a. Who provided this support?
   b. What specific activities have taken place? Example: planning meetings, trainings, data reviews, etc.

8. FOR MAINTENANCE ONLY: Are you aware that the APHIA contract is ending soon?

   IF NO, SKIP TO NEXT QUESTION
   IF YES, ASK QUESTIONS BELOW:

   a. Who told you about this?
   b. How will the support you receive from the APHIA program change under the new program?

      PROBE: HIV treatment, prevention, outreach, testing, above-site, workplanning, etc.

c. What is being planned for the switch between implementing partners and/or projects?

d. Will there be any breaks in support to your team or for [NAME OF SITE]? If so, how will you manage this?

e. How did you/your facility react to the switch in programs?

Effects of Geographic Prioritization

INTERVIEWER*:* Now I would like to ask you a series of questions facility services and performance at your facility.

9. How have clinical services at the facility changed since May 2017?
    a. Clinical changes:
        i. HIV clinical services: HIV testing, treatment, referrals
            1. ***Is CD4 testing being conducted?
        ii. Community outreach
        iii. Pediatric services
        iv. Non-HIV services: antenatal care, family planning, malaria, tuberculosis
            1. ***Are blood hematology tests being conducted?
    b. Are you aware of any shifts in patient patterns of care seeking – for example do you think patients are shifting away from facilities that transitioned during Geographic Prioritization and going to those that continue to receive implementing partner support?
        i. ***MARSABIT ONLY: Are you aware of information being spread on the radio and news? If so, how did this affect patient volume and care seeking at this facility?
    c. Why have these changes taken place?

        PROBE: changes related to transition or other contextual issue?
    d. How do you perceive these changes -do you think they are for the better or for the worse?
    e. How are these changes perceived by the staff? By the community?
    f. Did you anticipate any of these changes?
    g. Are there any plans to address these changes?
        i. If so, who will be responsible? E.g. county leadership, PEPFAR implementing partners, national government, other donors, etc.

10. How has the management and organization of the facility changed since May 2017?
    a. Health systems changes:
        i. Health workforce:
            1. Has the facility lost or gained staff?
            2. ***What has happened with expert patients? Have they been reinstated?

3. What differences have you seen between permanent staff and temporary staff, or the different categories of health staff?
4. How has staff turnover changed?
5. How has staff motivation or performance changed?

ii. Commodity supply – have there been problems with drug availability or costs of drugs?

1. ***Is the facility charging patients for drugs? If so, which drugs?

iii. How about Lab services? Are you able to offer clients the same array of tests? Has the turnaround on obtaining test results changed?

1. ***Is the facility charging patients for tests? If so, which tests?
2. ***Are you having difficulties transporting any of your lab samples?
   a. For Viral Load?
   b. For Early Infant Diagnosis (EID)?
   c. For Gene Expert?

iv. Budgets – any notable changes to budgets and expenditures for the facility?

v. Reporting – have you been able to maintain regular reporting to DHIS? Are you submitting any reporting to [IMPLEMENTING PARTNERS/PROJECTS]?

vi. Infrastructure

vii. Any changes to user fees? If so, for what services?

b. Any difference between how HIV and non-HIV services are managed or organized?

c. Why have these changes taken place?

PROBE: changes related to Geographic Prioritization or other contextual issue?

d. How do you perceive these changes, do you think they are for the better or for the worse?

e. How are these changes perceived or experienced by the facility staff?

f. Did you anticipate any of these changes?

g. Are there any plans to address these changes?

i. If so, who will be responsible? E.g. county leadership, PEPFAR implementing partners, national government, other donors, etc.

11. How has the facility performed overall since May 2017?

a. Has the facility been able to adopt new practices, like test and treat?

i. If so, was this easy or difficult to do?

ii. If not, why not? Any plans to do this in the future?

12. How has service coverage changed since May 2017?

a. Who has been the most affected as a result of transition? E.g. HIV patients, non-HIV patients; patients living far away from the facility etc.
b. In what way have patients been affected? E.g. longer waiting times, higher out of pocket costs for HIV services/medicines/tests; etc.
c. Why has this happened?
d. Have there been effects on all the HIV services offered? E.g. PMTCT, ART, prevention, testing, etc.
    i. Why or why not?
e. How has coverage for non-HIV services, like maternal and child health been affected?
    i. Why has this happened?
f. FOR CENTRAL SUPPORT ONLY:
    i. How much of the change in coverage would you think is related to the Geographic Prioritization/transition?

13. How has the county health office adapted its operations since May 2017? Please explain.
    a. How has your relationship with the county health office changed?
        i. ***Has the county assisted you with any work planning or budgeting?
    b. How has your relationship with the national level changed?
    c. How has your relationship with [IMPLEMENTING PARTNER] changed?
    d. Have you collaborated with other facilities? E.g. referrals, commodities, staff.
    e. Describe any major challenges you were facing during this period.
    f. FOR CENTRAL SUPPORT ONLY:
        i. How much of these changes is related to the Geographic Prioritization?

14. FOR CENTRAL SUPPORT ONLY: In your view what else should have been done prior to the transfer in order to help with the transition process, which was not done?

    PROBE ON DIFFERENT STAKEHOLDERS: county/district health office, national government, PEPFAR, implementing partners, local government team, etc.

15. Is there anything else significant about recent changes at this facility that we should know about?

Thank you for your time and contribution

Project SOAR – Longitudinal Case Studies of PEPFAR Geographic Prioritization

SEMI-STRUCTURED INTERVIEW GUIDE – LOCAL GOVERNMENT OFFICIALS (COUNTY)

ROUND 2

INTRODUCTION

Thank you for agreeing to meet us.

We are conducting an assessment of PEPFAR's geographic prioritization process; that is, the process through which sites have transitioned from PEPFAR support to central support. We are interested in the processes that took place before transition to prepare and after transition under central support. Our goal is to provide practical information to local and national government, PEPFAR and other partners about how the transition process took place and whether it has affected how services are delivered.

As part of the overall evaluation, we are conducting case studies of facilities that are transitioning from PEPFAR support. In your county we are studying [NAME OF SITE] and would like to ask you some questions regarding the transfer process for this particular facility, but we also want you to reflect on the transition process that all facilities are going through. We conducted a first round of interviews earlier this year, and we have returned to understand how things have changed since then.

Name of Organization ______________________

Your name ______________________

Designation ______________________

Work Area ______________________

Postal address ______________________

Telephone ______________________

E-mail address ______________________

OBTAIN INFORMED CONSENT

NOTE TO INTERVIEWER: This is a guide to the interview. You should cover ***all the main numbered questions*** *in this interview form. You should use the probes selectively, according to the type of knowledge that the respondent conveys, and what you have already found out from documents and other interviews.*

INTERVIEW QUESTIONS

***INTERVIEWER**: If respondent was interviewed before and position has not changed, skip Q1 and Q2. If respondent was not interviewed before or if role has changed, ask Q1 and Q2*

1. Can you tell me a little about your current role, and how familiar you are with this facility (NAME SITE)?

***INTERVIEWER**: If the respondent does not seem at all familiar with the case study facility, then please ask if there is someone else who is more familiar with the facility whom you could talk to.*

2. Are you familiar with the recent transition of this site (NAME SITE) and/or county from [PEPFAR OR IMPLEMENTING PARTNER] support to government?

   PROBE: Have you had any recent changes in how [IMPLEMENTING PARTNER] has supported the facility?

   a. Were you involved at all in this transition process at this facility?

***INTERVIEWER**: If the respondent does not seem at all familiar with transition, then please ask if there is someone else who is more familiar with transition whom you could talk to.*

3. What is the current status of [NAME OF SITE] in regards to PEPFAR's Geographic Prioritization? Is this facility still receiving direct PEPFAR support?

   ***INTERVIEWER:** Share the status reported in previous data collection rounds for this facility.*

   a. *IF THE RESPONSE IS DIFFERENT FROM STATUS REPORTED IN ROUND 1:* When did this change happen? Why?
   b. Who, if anyone, explained the process to you and your colleagues?

Support to Facilities

INTERVIEWER: Now I'm going to ask you about how support for [NAME OF SITE] has changed since around May 2017.

4. Please describe all of the current projects and/or partners that have been supporting [NAME OF SITE] since May 2017.

   PROBE: PEPFAR implementing partners/projects, non-PEPFAR implementing partners/projects

   a. What support are they providing to facilities? Examples: staff hiring and salaries, commodities, training, funding, support for reporting, patient incentives, etc.
   b. When did each project/implementing partner start?
   c. Any support for non-HIV services, like maternal and child health? E.g. antenatal care, immunizations, malaria, etc.

5. Please tell me about all of the projects/implementing partners that have stopped supporting [NAME OF SITE] since May 2017.

PROBE: PEPFAR implementing partners/projects, non-PEPFAR implementing partners/projects

a. When did they stop their support?
b. Why did they stop their support?
c. What support were they providing?
d. What happened to the support after they left?

6. How are activities at [NAME OF SITE] supported now? By whom?
    a. Who provides this support? E.g. staff hiring and salaries, commodities, training, funding, support for reporting, patient incentives, laboratory services etc.

       PROBE: county, national, other projects, NGOs, civil society, etc.

    b. Any support for non-HIV services, like maternal and child health? E.g. antenatal care, immunizations, malaria, etc.?
    c. What kind of support has the facility received from government since May 2017?
    d. Any additional areas of support that are new or had not been provided before?
    e. How is this arrangement different from how activities were supported before May 2017?
    f. What agreements have been put in place between county government and [IMPLEMENTING PARTNER] regarding support to [NAME OF SITE]?
        i. Is this a new agreement? What brought it about?
        ii. What does this agreement cover?

7. What support has <u>your team</u> provided to [NAME OF SITE] since May 2017?
    a. What specific activities have been done? E.g. other resources, meetings, trainings, data reviews, etc.
    b. Who else has provided support to help [NAME OF SITE]?
    c. Is this similar for other facilities across the county? Why/why not?
    d. How has your relationship with [IMPLEMENTING PARTNER[S)] changed since May 2017?
    e. *FOR CENTRAL SUPPORT ONLY:*
        i. How have you filled the gaps left by departing implementing partner support?
        ii. How have you involved facility staff and patients in managing the loss of support through the transition?

Support for County

Now I'm going to ask you about how support for [NAME OF COUNTY] has changed since PEPFAR's geographic prioritization.

8. Please describe all of the current partners that support HIV services in the county.

PROBE: PEPFAR implementing partners/projects, non-PEPFAR implementing partners/projects, national government, MOH, regional/county government, NGOs/civil society, etc.

a. How long has each of these partners been working here?

b. What support are they providing to facilities? Examples: staff hiring and salaries, commodities, training, funding, support for reporting, patient incentives, laboratory etc.

    i. How does this support vary by type or size of facility?

c. When did the support from these different partners start?

9. Are you aware of any organizations that have stopped supporting the county since May 2017?

    a. IF so, when did they stop their support?

    b. Why did they stop their support?

    c. What support were they providing?

    d. What happened to that support after they left?

10. Please tell me about the types of support <u>your team</u> is currently receiving. E.g. facilitation for meetings, supplies, transport or fuel for transport, staff hiring, training, support for reporting etc.

    PROBE: From whom? Which projects/implementing partners, national government, other county, civil society, etc.

    a. Was this support targeted for a particular health area (e.g. HIV service management and planning, maternal and child health)?

    b. How is this support different from the support [IMPLEMENTING PARTNER] provided to [COUNTY] since May 2017? Why?

    c. ***Have you received support for planning and budgeting? If so, who provided this support?

11. How much of this support is new since May 2017?

    PROBE: new partners, new activities, changes between partners and government, etc.

    a. What brought about this new support? E.g. newly identified need, new contracts issued, etc.

    b. Do you anticipate any new partners to start providing support within the next 6 months?

12. FOR MAINTENANCE ONLY: Are you aware that the APHIA contract is ending soon?

    IF NO, SKIP TO NEXT QUESTION
    IF YES, ASK QUESTIONS BELOW:

    a. Who told you about this?

    b. How will the support you receive from the APHIA program change under the new program?

PROBE: HIV treatment, prevention, outreach, testing, above-site, workplanning, etc.

c. What is being planned for the switch between implementing partners and/or projects?

d. Will there be any breaks in support to your team or for [NAME OF SITE]? If so, how will you manage this?

e. How did your team react to the switch in programs?

Effects of Geographic Prioritization

INTERVIEWER*:* Now I would like to ask you a series of questions facility services and performance at [NAME OF SITE].

13. How have clinical services changed at [NAME OF SITE] since May 2017?

    a. Clinical changes:

        i. HIV clinical services: HIV testing, treatment, referrals

            1. ***Is CD4 testing being provided at [NAME OF SITE]?

        ii. Community outreach

        iii. Pediatric services

        iv. Non-HIV services: family planning, malaria, tuberculosis

            1. ***Are hematology tests being provided at [NAME OF SITE]?

    b. Are you aware of any shifts in patient patterns of care seeking – for example do you think patients are shifting away from facilities that transitioned during Geographic Prioritization and going to those that continue to receive Implementing Partner support?

        i. ***MARSABIT ONLY: Are you aware of information on transition being spread by the radio and local news by expert patients? If so, what was the impact of this on the facility and patient care seeking?

    c. Why have these changes taken place?

       PROBE: changes related to Geographic Prioritization or other contextual issue?

    d. How do you perceive these changes, do you think they are for the better or for the worse?

    e. How are these changes perceived by the staff and community?

    f. Did you anticipate any of these changes?

    g. Are there any plans to address these changes?

        i. If so, who will be responsible? E.g. county/district leadership, PEPFAR implementing partners, national government, other donors, etc.

14. How has the organization and management of [NAME OF SITE] changed since May 2017?

    a. Health systems changes:

        i. Health workforce

            1. Has [NAME OF SITE] lost or gained staff?
            2. Any changes to motivation or performance?
            3. ***What has happened with expert patients? Have they been reinstated?
        ii. Commodity supply – have there been problems with drug availability or costs of drugs at [NAME OF SITE]?
            1. ***Is [NAME OF SITE] charging patients for any drugs? If so, which drugs?
        iii. Lab services – have there been problems with availability of tests or sample networking at [NAME OF SITE]?
            1. ***Is [NAME OF SITE] charging patients for any tests? If so, which tests?
            2. ***Is [NAME OF SITE] experiencing challenges with transporting lab samples?
                a. For Viral Load?
                b. For Early Infant Diagnosis (EID)?
                c. For Gene Expert?
        iv. Budgets – any notable changes to budgets and expenditures for [NAME OF SITE]?
        v. Reporting to DHIS – has [NAME OF SITE] been able to maintain regular reporting to DHIS?
        vi. Infrastructure
        vii. Any difference between HIV and non-HIV services?
    b. Why have these changes taken place?

       PROBE: changes related to Geographic Prioritization or other contextual issue?
    c. How do you perceive these changes, do you think they are for the better or for the worse?
    d. How are these changes perceived or experienced by the health workers?
    e. Did you anticipate any of these changes?
    f. Are there any plans to address these changes?

15. How has [NAME OF SITE] performed overall since May 2017?
    a. Has the facility been able to adopt new practices, like test and treat?
        i. If so, was this easy or difficult to do?
        ii. If not, why not? Any plans to do this in the future?

16. How has service coverage at [NAME OF SITE] changed since May 2017?
    a. Why has this happened?
    b. Have there been effects on coverage for all the HIV services offered? E.g. PMTCT, ART, prevention, testing, etc.

        i. Why or why not?

   c. How has coverage for non-HIV services, like maternal and child health, been affected?

        i. Why has this happened?

   d. FOR CENTRAL SUPPORT ONLY:

        i. How much of the coverage changes is related to the Geographic Prioritization?

17. How has the county health office adapted its operations since May 2017?
    a. What changes have you made in the way you operate? For example, changes to reporting, staffing at the district/county level, etc.

    PROBE: lab collections and deliveries, reporting support, paying for trainings.

INTERVIEWER*: Ask Q18 and Q19 ONLY for facilities that transitioned to Central Support.*

18. How has the county health system responded to the transfer of [NAME OF SITE] away from PEPFAR support since May 2017? Please explain.
    a. How has your relationship with the facilities changed?
    b. How has your relationship with the national level changed?
    c. How has your relationship with [IMPLEMENTING PARTNER] changed?
    d. Have you collaborated with other county/district governments? E.g. referrals, commodities, staff.
    e. Describe any major challenges you were facing during this period.

19. In your view what else should have been done prior to the transfer of [NAME OF SITE] in order to help with the transition process, which was not done?

    PROBE ON DIFFERENT STAKEHOLDERS: national government, PEPFAR, implementing partners, local government team, etc.

20. How does what you have observed at [NAME OF SITE] compare to other facilities in your county?
    a. How does this vary between public facilities and private facilities, both for profit and not-for-profit?

21. Is there anything else significant about recent changes at this facility that we should know about?

Thank you for your time and contribution

****FOLLOW-UP →** Does this county have a county HIV committee? Who is a member?

Project SOAR – Longitudinal Case Studies of PEPFAR Geographic Prioritization

<u>SEMI-STRUCTURED INTERVIEW GUIDE – PEPFAR IMPLEMENTING PARTNER – MAINTENANCE (ROUND 2)</u>

INTRODUCTION

Thank you for agreeing to meet us.

We are conducting an assessment of PEPFAR's geographic prioritization process; that is, the process through which sites have transitioned from PEPFAR support to central support. We are interested in the processes that took place before transition to prepare and after transition under central support. Our goal is to provide practical information to local and national government, PEPFAR and other partners about how the transition process took place and whether it has affected how services are delivered.

As part of the overall evaluation, we are conducting case studies of facilities that are transitioning from PEPFAR support and some that are being sustained. We are studying [NAME OF SITE] and would like to ask you some questions regarding this particular facility, but we also want you to reflect on the transition process that all facilities are going through. We conducted a first round of interviews earlier this year, and we have returned to understand how things have changed since then.

Name of Organization ______________________________

Your name ______________________________

Designation ______________________________

Work Area ______________________________

Postal address ______________________________

Telephone ______________________________

E-mail address ______________________________

OBTAIN INFORMED CONSENT

NOTE TO INTERVIEWER: This is a guide to the interview. You should cover ***all the main numbered questions*** *in this interview form. You should use the probes selectively, according to the type of knowledge that the respondent conveys, and what you have already found out from documents and other interviews.*

INTERVIEW QUESTIONS

***INTERVIEWER**: If respondent was interviewed before and position has not changed, skip Q1.*

1. Can you tell me a little about your current role, and how familiar you are with [NAME OF SITE]?

***INTERVIEWER**: If the respondent does not seem at all familiar with the case study facility, then please ask if there is someone else who is more familiar with the facility whom you could talk to.*

2. **New respondents only:** Are you familiar with the recent PEPFAR policy through which certain districts and facilities transition away from [PEPFAR OR IMPLEMENTING PARTNER] support?
    a. Were you involved at all in this transition process?

***INTERVIEWER**: If the respondent does not seem at all familiar with transition, then please ask if there is someone else who is more familiar with transition whom you could talk to.*

3. What is the current status of [NAME OF SITE] in regards to PEPFAR's Geographic Prioritization? Is this facility still receiving direct PEPFAR support?

***INTERVIEWER:** Share the status reported in previous data collection rounds for this facility*

    a. *IF THE RESPONSE IS DIFFERENT FROM STATUS REPORTED IN ROUND 1:* When did this change happen? Why?

Support for Services

***INTERVIEWER**:* Now I'm going to ask you about the current status of PEPFAR support for services.

4. What kind of support, if any, has the facility received from you <u>since May 2017</u>? (E.g. staff hiring and salaries, commodities, training, funding, support for reporting, patient incentives, etc.)

    PROBE: Which IP providing which support, frequency, etc.

    a. Has the support given changed since then?
    b. Has the facility lost any support from you? Why did they lose support?
    c. Any additional areas of support that had not been provided before?

5. Who else currently works with or supports [NAME OF SITE] that you are aware of? E.g. county health offices, national AIDS agencies, PEPFAR supported partners, non-PEPFAR supported partners, civil society, etc.
    a. What support do they provide?

    PROBE: HIV service delivery support, HIV outreach, MCH or non-HIV support

    b. When did this support start? Is it ongoing?
        i. IF SUPPORT IS NEW: What prompted the start of this support?
        ii. Are you aware of when support is supposed to end? Why?

c. Has your organization collaborated with these organizations to provide support to [NAME OF SITE]?

6. Are you aware whether any other organization have STOPPED providing support to [NAME OF SITE] in the past year?
    a. If so, who and why did they stop?

7. How will the support for [NAME OF SITE] change when the APHIA contracts end?

    PROBE: HIV treatment, prevention, outreach, testing, above-site, workplanning, etc.

    a. How will the process of switching between implementing partners and/or contracts go?
        i. Will there be any breaks in support to counties? If so, how will this be managed?
    b. How did counties and facilities react to the switch in programs?

8. How are non-HIV services that used to be offered through the APHIAs provided now?
    a. Are non-HIV services also prioritized geographically?
        i. If so, which services are prioritized and why?
    b. How does this support vary by investment category?

Effects of Geographic Prioritization

INTERVIEWER*:* Now I would like to ask you a series of questions about facility services and performance at [NAME OF SITE].

9. How have clinical services at the facility changed since May 2017?
    a. Clinical changes:
        i. HIV clinical services: HIV testing, treatment, referrals
            1. ***Is [NAME OF SITE] providing CD4 tests?
        ii. Community outreach
        iii. Pediatric services
        iv. Non-HIV services: family planning, malaria, tuberculosis
            1. ***Is [NAME OF SITE] providing hematology tests?
    b. Are you aware of any shifts in patient patterns of care seeking – for example do you think patients are shifting away from facilities that transitioned during Geographic Prioritization and going to those that continue to receive implementing partner support?
    c. Why have these changes taken place?

PROBE: changes related to PEPFAR geographic prioritization or other contextual issue?

d. How do you perceive these changes: do you think they are for the better or the worse?

e. How are these changes perceived by the facility staff and community?

f. Did you anticipate any of these changes?

g. Are there any plans to address these changes?

    i. If so, who will be responsible? E.g. county leadership, PEPFAR implementing partners, national government, other donors, etc.

10. How has the organization and management of the facility itself changed since May 2017?

    a. Health systems changes:

        i. Health workforce- has [NAME OF SITE] lost or gained staff? Any changes to motivation or performance?

        ii. Commodity supply – have there been problems with drug availability or costs of drugs at [NAME OF SITE]?

            1. ***Are you aware of [NAME OF SITE] charging for any drugs? If so, which drugs?

        iii. Lab services – are you aware of any problems related to the array of tests offered to clients? The turnaround time for obtaining test results?

            1. ***Are you aware of [NAME OF SITE] charging for any tests? If so, which tests?

            2. ***Is [NAME OF SITE] experiencing challenges with transporting lab samples?

                a. For Viral Load?

                b. For Early Infant Diagnosis?

                c. For Gene Expert?

        iv. Budgets – any notable changes to budgets and expenditures for [NAME OF SITE]?

        v. Reporting to DHIS – has [NAME OF SITE] been able to maintain regular reporting to DHIS?

        vi. Infrastructure

        vii. Any difference between HIV and non-HIV services?

    b. Why have these changes taken place?

    PROBE: changes related to PEPFAR geographic prioritization or other contextual issue?

    c. How do you perceive these changes: do you think they are for the better or the worse?

    d. How are these changes perceived or experienced by the health workers?

    e. Did you anticipate any of these changes?

    f. Are there any plans to address these changes?

    i. If so, who will be responsible? E.g. county leadership, PEPFAR implementing partners, national government, other donors, etc.

11. How has the facility performed overall <u>since</u> May 2017?
    a. Has the facility been able to adopt new practices, like test and treat?
        i. If so, was this easy or difficult to do?
        ii. If not, why not? Any plans to do this in the future?

12. How has service coverage changed since May 2017?
    a. Why has this happened?
    b. Have there been effects on coverage for all the HIV services offered? E.g. PMTCT, ART, prevention, testing, etc.
        i. Why or why not?
    c. How has coverage for non-HIV services, like maternal and child health, been affected?
        i. Why has this happened?

13. How does what you have observed at [NAME OF SITE] compare to other facilities that you have supported?

14. How has your relationship with the county health office changed since May 2017? Please explain.
    a. What kind of support has the county received from you or other implementing partners <u>since May 2017</u>, if any? (E.g. staff hiring and salaries, commodities, training, funding, support for reporting, patient incentives, etc.)
        i. ***How have you supported the county with work planning and budgeting?

    PROBE: Which IP providing which support, frequency, etc.

    b. Does the county receive any funding from your organization?
        i. If so, do you know what this covers?
        ii. Is this funding new since May 2017?

15. How has your organization changed how it operates since May 2017?
    a. Have you faced any internal challenges, such loss of funding for particular staff positions, and how did you deal with these?
    b. How has your relationship with PEPFAR changed?

16. Broadly speaking, what is your general impression about the Geographic Prioritization process, and how this went?
    a. Has your opinion changed since May 2017?
    b. What challenges did facilities, local government teams, and implementing partners experience in terms of the Geographic Prioritization and how it was implemented? Please explain.
    c. What else should have been done in order to facilitate the Geographic Prioritization process, which was not done?
    d. How do you think the relationship between county and PEPFAR IP support will change in the next six months? In the next year?
    e. What do you think will happen when your project ends?

17. Is there anything else significant about changes at this facility since May 2017 related to how the geographic prioritization process has occurred that we should know about?

Thank you for your time and contribution

Project SOAR – Longitudinal Case Studies of PEPFAR Geographic Prioritization

FOCUS GROUP GUIDE – PATIENTS (ROUND 2)

INTRODUCTION

Thank you for agreeing to meet us.

We are studying the process through which some support to facilities is transferred from PEPFAR to the local government. We are hoping to provide practical information to local and national government, PEPFAR and other partners about how the transfer process may have taken place and whether it has affected how services are delivered. We conducted a first round of interviews earlier this year, and we have returned to understand how things have changed since then.

Name of facility	---
Names of community members	---

Postal address of the facility	---

Telephone of facility	---
E-mail address of facility	---

OBTAIN INFORMED CONSENT

NOTE TO INTERVIEWER: Please cover ***all the main numbered questions*** *in the interview guide. Probes should be used selectively based on the kind of knowledge that the respondents convey and what you have already found from documents relating to transition and other interviews.*

QUESTIONS

1. Please can you introduce yourself and tell me how long have you been attending this facility?

***INTERVIEWER**: Please ask each individual their name, age and how far they live from the facility.*

***INTERVIEWER**: Ask Q2 <u>ONLY</u> in facilities that transitioned to Central Support.*

2. Are you aware that this facility has recently shifted away from receiving support from [PEPFAR/IMPLEMENTING PARTNER]?

***INTERVIEWER**: Ask remaining questions in all facilities.*

3. What organizations support this facility now? E.g. county government, MOH, APHIA, etc.
    a. What kinds of support do they provide for patients like you? E.g. refreshments, support to meetings, family support
    b. Have these changed at all since May 2017? E.g. new organizations started, old organizations left, other changes.

4. What <u>changes to HIV services</u> have you noticed since May 2017?
    a. Was there any interruption in services?
    b. Do you see any changes in the <u>types of services</u> that the facility provides? If so, please explain.

       PROBE: treatment, prevention, outreach.
    c. Do you see any changes in the <u>quality of services</u> that the facility provides? If so, please explain.
    d. Can you explain why these changes have taken place?
    e. Have there been any changes to the drugs that are available at this facility?
        i. ***Have you had to pay for any drugs? If so, which drugs?
    f. Have there been any changes in the types of laboratory tests that are available? How has the turnaround on obtaining test results changed?
        i. ***Is the clinic still providing CD4 testing?
        ii. ***Have you had to pay for any tests? If so, which tests?
    g. How have costs at this facility changed? E.g. For services, drugs or tests.
    h. ***MARSABIT ONLY: Have you heard from other patients or on the radio of HIV services at Tumaini ending?
        i. What exactly did you hear?
        ii. When did you hear this? From whom?
        iii. How did you react? Did you continue to come to Tumaini?

5. What <u>changes to non-HIV services, like maternal and child health</u>, have you noticed since May 2017?
    a. Was there any interruption in services?
    b. Do you see any changes in the <u>types of services</u> that the facility provides? If so, please explain.

       PROBE: ANC, immunizations, etc.
    c. Do you see any changes in the <u>quality of services</u> that the facility provides? If so, please explain.
    d. Can you explain why these changes have taken place?
    e. Have there been any changes to the drugs that are available at this facility?
        i. ***Have you had to pay for any drugs? If so, which drugs?
    f. Have there been any changes in the types of laboratory tests that are available? How has the turnaround on obtaining test results changed?
        i. ***Is the clinic still providing blood tests?
        ii. ***Have you had to pay for any tests? If so, which tests?
    g. How have costs at this facility changed? E.g. For services, drugs or tests.

6. Since May 2017, have there been changes to <u>the way that services are delivered</u> at this facility? E.g. changes in staffing, the way services were provided etc.
    a. Did the facility makes any changes in the way it operates?
    b. Do you think the staff are as motivated as they were before?
    c. Has there been much turnover of staff?
    d. ***What is the situation with expert clients at this facility?
        i. IF GONE AND THEN RETURNED: When did they come back?

7. How do you feel about the changes at the facility recently?
    a. How have you coped with these changes?
        i. How have other people you know coped with these changes?
    b. Would you consider switching to another facility to receive your services? Why/why not?
        i. If so, where would you go? Why?
        ii. If so, do you think it would be harder or easier to get the care you want? Why/why not?

8. Do you think there is anything else significant about recent changes taking place at this facility that we should know about?

Many thanks for your help and time.
